# Whole‐Genome Promoter Profiling of Plasma DNA Exhibits Diagnostic Value for Placenta‐Origin Pregnancy Complications

**DOI:** 10.1002/advs.201901819

**Published:** 2020-02-18

**Authors:** Zhiwei Guo, Fang Yang, Jun Zhang, Zhigang Zhang, Kun Li, Qi Tian, Hongying Hou, Cailing Xu, Qianwen Lu, Zhonglu Ren, Xiaoxue Yang, Zenglu Lv, Ke Wang, Xinping Yang, Yingsong Wu, Xuexi Yang

**Affiliations:** ^1^ Institute of Antibody Engineering School of Laboratory Medicine and Biotechnology Southern Medical University Guangzhou 510515 China; ^2^ Department of Obstetrics and Gynecology Nanfang Hospital Southern Medical University Guangzhou 510515 China; ^3^ Department of Obstetrics The Third Affiliated Hospital of Sun Yat‐sen University Guangzhou 510630 China; ^4^ Department of Pathology Cangzhou People's Hospital Cangzhou 061000 China

**Keywords:** cell‐free DNA, early prediction, pregnancy complications, promoter profiling, whole‐genome sequencing

## Abstract

Placenta‐origin pregnancy complications, including preeclampsia (PE), gestational diabetes mellitus (GDM), fetal growth restriction (FGR), and macrosomia (MA) are common occurrences in pregnancy, resulting in significant morbidity and mortality for both mother and fetus. However, despite their frequency, there are no reliable methods for the early diagnosis of these complications. Since cfDNA is mainly derived from placental trophoblasts and maternal hematopoietic cells, it might have information for gene expression which can be used for disease prediction. Here, low coverage whole‐genome sequencing on plasma DNA from 2,199 pregnancies is performed based on retrospective cohorts of 3,200 pregnant women. Read depth in the promoter regions is examined to define read‐depth distribution patterns of promoters for pregnancy complications and controls. Using machine learning methods, classifiers for predicting pregnancy complications are developed. Using these classifiers, complications are successfully predicted with an accuracy of 80.3%, 78.9%, 72.1%, and 83.0% for MA, FGR, GDM, and PE, respectively. The findings suggest that promoter profiling of cfDNA may be used as a biological biomarker for predicting pregnancy complications at early gestational age.

## Graphical abstract

Cell free DNA (cfDNA) comprises a nucleosome footprint that carries information of its tissue of origin. Different pregnancy complications show distinct patterns of promoter profiling. Based on promoter profiling and machine learning methods, the prediction classifiers can forecast four types of pregnancy complications, such as macrosomia, fetal growth restriction, gestational diabetes, and preeclampsia at early gestational age.

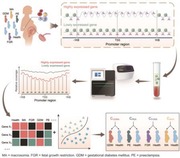

## 1 Introduction

Placenta‐origin pregnancy complications are common, with preeclampsia (PE) observed in 3–8%,^[^[qv: 1]^]^ gestational diabetes mellitus (GDM) in approximately 10%,^[^[qv: 2,3]^]^ fetal growth restriction (FGR) in 5–10%,^[^[qv: 4]^]^ and macrosomia (MA) in 3–15% of pregnancies.^[^[qv: 5,6]^]^ These complications often lead to adverse maternal and fetal outcomes during as well as subsequent pregnancies, including abnormal fetal development, thromboembolic complications, and an increased risk of diabetes for mothers and their offspring.^[^[qv: 7]^]^ Multivariate screening methods based on ultrasound examination and the quantification of diverse maternal urine and serum biomarkers have recently been proposed.^[^[qv: 8–11]^]^ Some researchers have developed metabolomic biomarkers for the early pregnancy prediction of preeclampsia.^[^[qv: 12,13]^]^ More reliable biomarkers for pregnancy complications are therefore needed to predict potential complications at early gestational age.

Elevated levels of cell‐free DNA (cfDNA) has been first reported in lupus patients^[^[qv: 14]^]^ and later in cancer patients.^[^[qv: 15]^]^ Since then, elevated cfDNA levels have been observed for a wide range of conditions, including pregnancy, infection, inflammation, ischemic stroke, myocardial infarction, and hemodialysis.^[^[qv: 16–20]^]^ Of these conditions, higher median maternal serum cfDNA concentrations have been reported in pregnancies with pregnancy complications, such as PE.^[^[qv: 21]^]^ Taken together, these observations indicate that cfDNA is a potential noninvasive biomarker of diverse diseases, including pregnancy‐related complications. However, it is necessary to distinguish the disease‐specific cfDNA patterns of different diseases before applying it to predict pregnancy complications in early pregnancy.

Plasma cfDNA fragments are released by apoptotic cells after enzymatic processing of chromatin. DNA that remains bound to nucleosomes is retained, whereas naked DNA regions between nucleosomes are digested.^[^[qv: 22–24]^]^ The resulting cfDNA, therefore, comprises a nucleosome footprint carrying information about its tissues of origin.^[^[qv: 25]^]^ For example, analysis of cfDNA fragment derived from cancers revealed that the promoter regions of active genes exhibited depleted coverage, which implied that less nucleosome‐binding occurred in these regions along with increased gene expression.^[^[qv: 26]^]^ In pregnant women, the majority of cfDNA is derived from maternal hematopoietic cells and placental trophoblasts.^[^[qv: 27]^]^ In addition, common pregnancy complications such as PE, GDM, FGR, and MA, have a root cause in the placenta, and involve the maternal immune system.^[^[qv: 20,28]^]^ Therefore, we hypothesized that cfDNA fragment distribution patterns may carry information regarding source tissues of origin, particularly placental trophoblasts and maternal hematopoietic cells, and that global profiling of cfDNA fragments in promoter regions can be used to identify biomarkers that can predict pregnancy complications.

**Figure 1:**
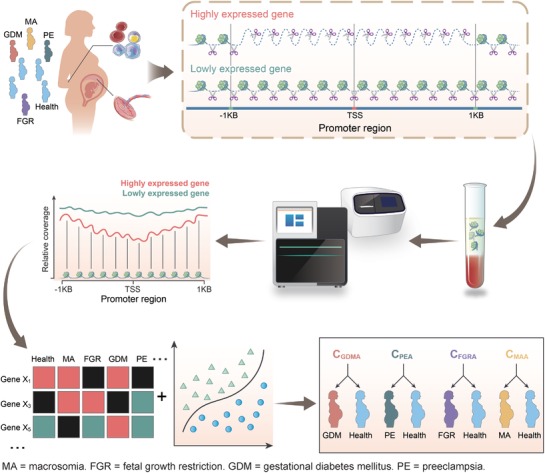
Schematic overview of predicting pregnancy complications. During pregnancy, the plasma cell free DNA (cfDNA) is primarily derived from placental trophoblasts and maternal hematopoietic cells. Exposed DNA not bound to a nucleosome is digested, whereas nucleosome‐bound DNA escapes digestion and enters into maternal circulation. Therefore, cfDNA comprises a nucleosome footprint that carries information of its tissue of origin and could reflect its gene expression pattern. As pregnancy complications are closely related to dysfunction of the placenta and maternal immune system, the read coverage of cfDNA may be used to predict the occurrence of pregnancy complications. We used whole‐genome sequencing data of cfDNA derived from 2199 pregnancies to develop classifiers for predicting four pregnancy complications—macrosomia (MA), fetal growth restriction (FGR), gestational diabetes mellitus (GDM), and preeclampsia (PE)—at early gestational age. To show greater differences, all nucleosome in the promoter regions (−1 KB to +1 KB around the transcription start site [TSS]) of highly expressed genes are depleted; however, the nucleosome‐depleted region is usually found within the nucleosome upstream of the TSS.

Here, we carried out a large‐scale, retrospective study using whole‐genome sequencing of plasma cfDNA from pregnant women at three independent hospitals, which included data from 3200 pregnant women (**Figure** **1**). According to their follow‐up results, 2199 participants (including 578 women with pregnancy complications and 1621 controls) were selected for promoter profiling analysis. Specific promoter profiling was found for MA, FGR, GDM, and PE. We then applied logistic regression to develop classifiers that could predict the occurrence of each complication. Using these classifiers, MA, FGR, GDM, and PE were successfully predicted with an accuracy of 80.3% (C_MA‐A_), 78.9% (C_FGR‐A_), 72.1% (C_GDM‐A_), and 83.0% (C_PE‐A_), respectively. Our findings suggested that cfDNA coverage across certain promoter regions detected at early gestational age may be used to develop simple and precise methods for predicting placenta‐origin pregnancy complications.

## 2 Results

### 2.1 Read Depth in Promoter Regions of Plasma DNA Infers Gene Expression Levels in Maternal Blood and Placenta Tissues

Read depths in the promoter regions detected using plasma cfDNA reflect the promoter activity of their respective genes in the tissues of origin and are negatively correlated with gene expression.^[^[qv: 26]^]^ The transcriptional activity of genes varies according to nucleosome occupancy at promoter regions, with decreased occupancy at the primary transcription start site (pTSS, defined as the region ranging from −1 to +1 KB around the transcriptional start site) of the active genes. Decreased nucleosome occupancy also leads to increased accessibility by DNA nucleases. As most of cfDNA in pregnancies was derived from maternal blood cells and placenta, we want to confirm whether the coverage of plasma cfDNA in promoter regions could reflect gene expression patterns of placenta and maternal blood. We carried out whole genome sequencing of cfDNA in 300 healthy pregnancies (Table S2, Supporting Information) to obtain promoter profiling on the read depths at the pTSS. In addition, the gene expression profiles of the placenta and maternal blood from healthy pregnancies were downloaded from Gene Expression Omnibus (GEO).

**Figure 2:**
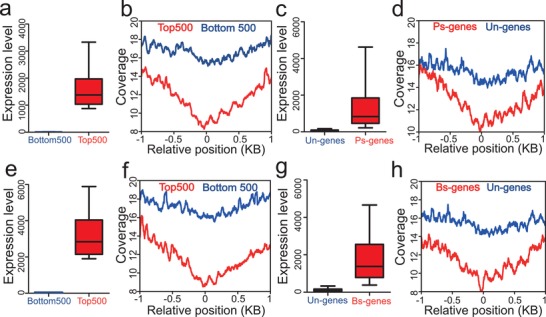
Promoter read depth patterns of highly and lowly expressed genes. a) Mean expression levels of the 500 most‐ (Top500, red) and least‐expressed (Bottom500, blue) genes in the placenta. b) Promoter coverage patterns for the 500 most‐ (Top500, red line) and least‐expressed (Bottom500, blue line) genes in the placenta. c) Mean expression levels of the placenta‐specific (Ps‐genes, red) and unexpressed (Un‐genes, blue) genes in the placenta. d) Promoter coverage patterns for the placenta‐specific (Ps‐genes, red) and unexpressed (Un‐genes, blue) genes in placenta. e) Mean expression levels of the most‐ 500 (Top500, red) and least‐expressed (Bottom500, blue) genes in the maternal blood. f) Promoter coverage patterns for the most‐ (Top500, red line) and least‐expressed (Bottom500, blue line) genes in the maternal blood. g) Mean expression levels of the whole blood‐specific (Bs‐genes, red) and unexpressed (Un‐genes, blue) genes in the maternal blood. h) Promoter coverage patterns for the whole blood‐specific (Bs‐genes, red) and unexpressed (Un‐genes, blue) genes in the maternal blood. Additional details about tissue‐specific and unexpressed genes can be found in Method section.

By comparing cfDNA coverage at the pTSS for the most 500 highest and 500 lowest expressed genes in the placenta and maternal blood (**Figure** **2**a,e), we confirmed that gene expression levels had negatively correlation with the read depths across the promoter regions. Highly expressed genes in the placenta exhibited lower read depth, whereas lowly expressed genes exhibited greater read depth (Figure 2b). Similar patterns were also evident for genes with high or low expression levels in the maternal blood (Figure 2f). As merely approximately 10% of total cfDNA was derived from placenta with the rest majorly derived from maternal blood cells, the promoter profiling of placenta may be affected by their common genes. Therefore, we further compared promoter profiling of placenta and whole blood specific genes and unexpressed genes. And we obtained similar results (Figure 2c, d, g, and h). Since most of pregnancy complications were correlated with the dysfunctions of placenta and maternal immune system, these results suggested that cfDNA distribution may reflect expression levels in the tissues of origin in pregnant women, meaning that cfDNA coverage at gene promoters can be applied as a biomarker to predict pregnancy complications. Using the expression profiles of placenta and whole blood cells derived from pregnancies with complications (see Section 4), we also found that the top 500 of highly expressed genes showed low coverage in the promoter regions and the bottom 500 of lowly expressed genes showed high coverage in the promoter regions (Figure S1, Supporting Information).

### 2.2 Promoter Profiling of cfDNA Reveals Disease‐Associated Patterns

**Figure 3:**
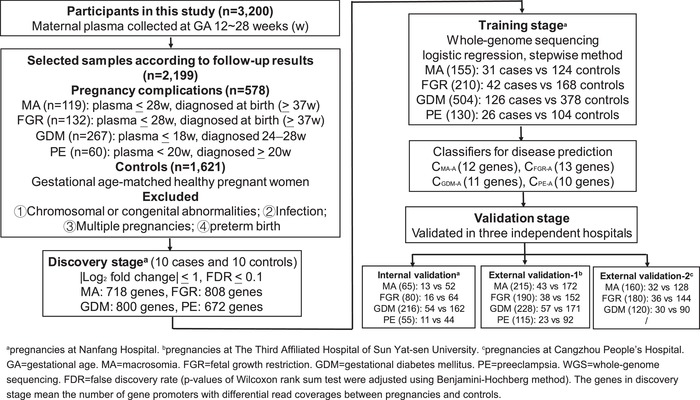
Pipeline used to develop classifiers for predicting pregnancy complications. To develop classifiers for predicting pregnancy complications, the plasma samples of pregnant women were collected before the diagnosis of pregnancy complications. The samples used for classifier construction were selected according to the gestational age of sampling and their follow‐up results. Additional details regarding the definition of pregnancy complications and corresponding controls are presented in Methods section. GA means gestational age. ^a,^ ^b^, and ^c^ indicate participants from the Nanfang Hospital of Southern Medical University, Third Affiliation Hospital of Sun Yat‐Sen University, and Cangzhou People's Hospital, respectively. Plasma cfDNA samples collected from Nanfang Hospital were analyzed using the Illumina sequencing platform. Plasma cfDNA samples collected from The Third Affiliation Hospital of Sun Yat‐Sen University and Cangzhou People′s Hospital were analyzed using the Ion Proton sequencing platform.

**Figure 4:**
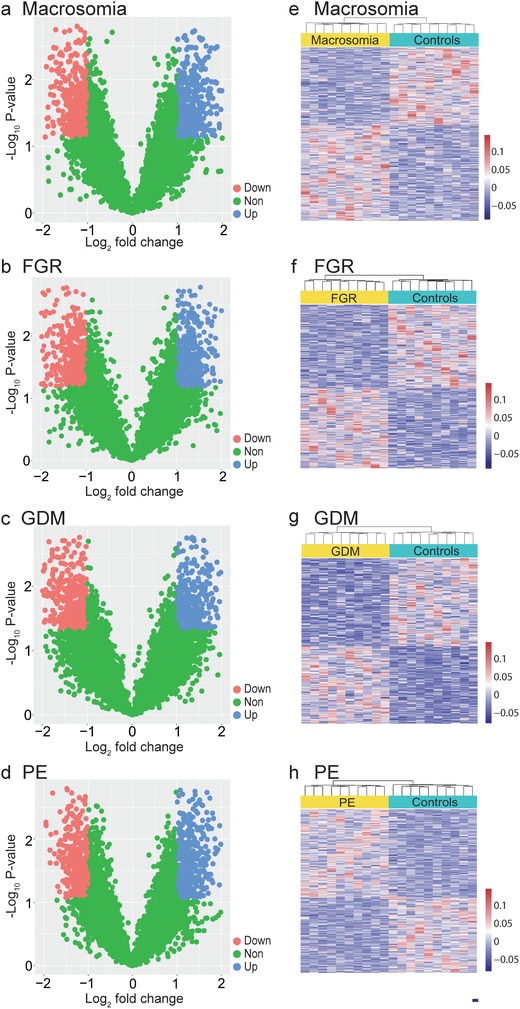
Gene transcripts with differential read coverages at primary TSS (pTSS). Volcano plots of gene transcripts with differential read coverages at the pTSS (|log_2_ fold change| ≥ 1 and false discovery rate [FDR] ≤ 0.1) at the pTSS detected using whole‐genome sequencing for a) macrosomia, b) FGR, c) GDM, and d) PE. The red, blue, and green dots indicate gene promoters though to be downregulated, upregulated, and exhibiting non‐differential coverage, respectively. Heat map of the z‐scores of promoters with differential read coverages for e) macrosomia, f) FGR, g) GDM, and h) PE.

To validate the potentials of cfDNA for predicting pregnancy complications, our study selected low coverage sequencing data of cfDNA derived from 2199 samples (119 MA, 132 FGR, 267 GDM, 60 PE and 1621 healthy controls) from three independent hospitals of China according to the gestational age of plasma collection and their follow‐up results (**Figure** **3**). For each complication, the gestational age of their controls was well matched in each cohort (Table S1, Supporting Information). There was no significant difference of the gestational age between cases and controls (*p*‐value: 0.76 for PE, 0.5 for GDM, 0.41 for FGR, 0.78 for MA, **Table** **1**). We developed a pipeline to search for effective classifiers (Figure 3), which included three stages: exploration of genes with differential promoter profiling (discovery stage), identification of classifiers (training stage) and validation of classifiers (validation stage). At the discovery stage, we first selected the low coverage whole‐genome sequencing data of cfDNA on ten complication cases and ten gestational age‐matched controls for each of the four pregnancy complications (MA, FGR, GDM, and PE). By comparing the promoter profiling of each pregnancy complication and their matched controls, we identified sets of genes with significant differential coverages (|Log_2_ fold change| ≥ 1 and FDR ≤ 0.1): 718 gene transcripts for MA, 808 for FGR, 800 for GDM, and 672 for PE, respectively (**Figure** **4**a–d and Table S6, Supporting Information). Next, we performed unsupervised clustering analysis on the coverages for these pregnancy complications. We found distinctive coverage patterns for MA (Figure 4e), FGR (Figure 4f), GDM (Figure 4g), and PE (Figure 4h), revealing distinctive coverage patterns for MA (Figure 4e), FGR (Figure 4f), GDM (Figure 4g), and PE (Figure 4h). The heatmaps show distinct patterns of promoter coverage between healthy pregnancies and pregnancies with complications (Figure 4e–h).

**Table 1:** Maternal and pregnancy characteristics of study pregnancies

	Macrosomia			FGR			GDM			PE		
	Case(*n* = 119)	Control(*n* = 476)	*p‐*value	Case(*n* = 132)	Control(*n* = 518)	*p‐v*alue	Case(*n* = 267)	Control(*n* = 801)	*p‐*value	Case(*n* = 60)	Control(*n* = 240)	*p‐*value
Gestational age at sampling (weeks)	16.9 ± 3.6	17.0 ± 3.6	0.78	18.3 ± 4.0	17.9 ± 3.7	0.41	15.9 ± 2.4	16.0 ± 2.6	0.5	17.9 ± 4.2	18.0 ± 4.2	0.76
Age (years)	33.1 ± 4.1	32.1 ± 4.7	0.052	31.1 ± 5.3	31.3 ± 5.1	0.67	34.0 ± 4.5	32.7 ± 4.8	1.6E‐03	34.1 ± 4.9	33.3 ± 4.4	0.17
BMI (kg m^−2^)	23.0 ± 2.3	21.2 ± 1.9	2.2E‐16	20.6 ± 3.1	21.5 ± 2.0	2.8E‐07	22.5 ± 2.8	21.3 ± 2.5	2.0E‐13	24.2 ± 3.5	21.3 ± 2.4	1.3E‐11
Weight gain (kg)	13.8 ± 4.3	13.9 ± 2.3	0.21	12.3 ± 3.2	13.7 ± 2.5	1.8E‐08	11.1 ± 3.1	13.4 ± 3.0	2.2E‐16	12.3 ± 3.5	13.0 ± 2.9	0.078
Baby weight (kg)	4.3 ± 0.6	3.3 ± 0.3	2.2E‐16	2.3 ± 0.3	3.3 ± 0.3	2.2E‐16	3.3 ± 0.5	3.2 ± 0.3	0.17	2.7 ± 0.6	3.2 ± 0.3	7.1E‐11
History of adverse pregnancy outcomes												
Yes	19	19	4.9E‐06	15	23	4.8E‐03	26	59	0.27	4	13	0.76*
No	100	457		117	495		241	742		56	227	

Data are mean ± standard deviation. Age = maternal age. BMI = pre‐pregnancy body mass index. Weight gain = weight gain during pregnancy. FGR = fetal growth restriction. GDM = gestational diabetes mellitus. PE = preeclampsia. Mann–Whitney *U*‐test was used for the comparison of continuous variables. χ^2^ test and Fisher exact test (*) were used for the comparison of categorical variables.

We searched the literature for the functional relevance of the top enriched pathways to the diseases, and found that each set of enriched pathways were associated with to the corresponding complication (Figure S2, Supporting Information and Table S7, Supporting Information). As one example, the PI3K‐Akt pathway regulates the expression of sFlt1, which is an important marker of PE in clinic.^[^[qv: 29]^]^

### 2.3 Identification and Validation of Classifiers Based on Genes with Differential Coverage at pTSS

To further validate the potentials of cfDNA for predicting complications, we applied more samples of whole‐genome sequencing of cfDNA to develop and validate prediction classifiers. At training stage, we focused on the gene transcripts with significant differential coverage at pTSS identified in the discovery stage. Using a logistic regression model and stepwise method for feature selection, a set of 12 genes, denoted by C_MA‐A_ (set‐A classifier)_,_ performed well as a predictor of MA (accuracy = 80.0%) and exhibited the largest AUC value (Table S8, Supporting Information). The probability of pregnancies with MA was calculated using C_MA‐A_ as follow:

(1) $$\begin{array}{c} \text{Logit}\left[p=\text{MA}\right]=2.180\;+\;0.605\;\times \;SMC3-1.204\;\times \;MASTL\\ +\;1.366\;\times \;CREM-1.295\;\times \;C1QTNF12-0.471\\ \times \;MLXIP\;-\;0.811\;\times \;MAP3K9-1.284\;\times \;IGSF6-1.347\\ \times \;APC2\;-\;0.504\;\times \;GPM6A\;+\;1.048\;\times \;TMEM128-0.057\\ \times \;NIPBL\;-\;1.652\;\times \;TMEM184A \end{array}$$

**Figure 5:**
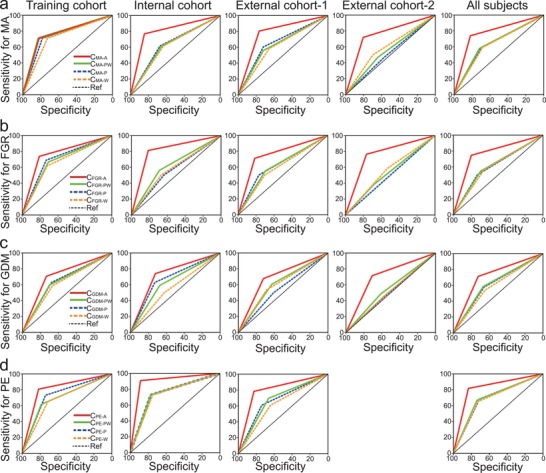
Performance of classifiers in predicting pregnancy complications. Receiver operating characteristic (ROC) curves for predicting a) macrosomia, b) FGR, c) GDM, and d) PE occurrences in the training, internal, and external cohorts for set‐A classifiers based on gene promoters with differential read coverages—C_MA‐A_, C_FGR‐A_, C_GDM‐A_, and C_PE‐A_; set‐P classifiers based on the promoter profiling of placenta‐specific genes—C_MA‐P_, C_FGR‐P_, C_GDM‐P_, and C_PE‐P_; set‐W classifiers based on the promoter profiling of whole blood‐specific genes—C_MA‐W_, C_FGR‐W_, C_GDM‐W_, and C_PE‐W_; and set‐PW classifiers based on the combination of placenta‐ and whole blood‐specific genes—C_MA‐PW_, C_FGR‐PW_, C_GDM‐PW_, and C_PE‐PW_. The *X*‐ and *Y*‐axes indicate classifier sensitivity and specificity, respectively.

Where *SMC3*, *MASTL*, *CREM*, *C1QTNF12*, *MLXIP*, *MAP3K9*, *IGSF6*, *APC2*, *GPM6A*, *TMEM128*, *NIPBL*, and *TMEM184A* are genes in the C_MA‐A_. In this equation, each gene was represented by a value of 1 when the normalized gene promoter coverage was higher than the corresponding cutoff (Table S9, Supporting Information); otherwise, the gene was represented by 0. Then the p‐value was calculated by logit transformation. If the p‐value was higher than the corresponding threshold (Table S13, Supporting Information), the pregnancy was predicted to have MA, otherwise the pregnancy was predicted to not have MA. In the training cohort, C_MA‐A_ had an AUC of 0.766 with a 95% confidence interval (95% CI of 0.678–0.854), which was used to determine whether individuals would develop MA or not. The accuracy of C_MA‐A_ was 80.0%, with a sensitivity of 71.0% and specificity of 82.3% in the training cohort (**Figure** **5**; Table S8, Supporting Information). Consistent with the results of the training cohort, the internal validation cohort and two external cohorts produced AUCs of 0.817 (0.689–0.945), 0.791 (0.721–0.861), and 0.762 (0.675–0.848), respectively, for the C_MA‐A_ (Figure 5; Table S8, Supporting Information).

For the other three pregnancy complications, the procedures for classifier construction and validation were similar to those used for MA (C_MA‐A_). For FGR, a set with 13 genes, denoted by C_FGR‐A_, performed well as predictor of FGR (accuracy = 79.5%; AUC = 0.774) during the training (Figure 5; Table S10, Supporting Information). The internal cohort and two external validation cohorts for C_FGR‐A_ produced AUCs of 0.813 (0.703–0.922), 0.763 (0.684–0.843), and 0.765 (0.684–0.845), respectively (Figure 5; Table S8, Supporting Information). For GDM, a set of 11 genes, denoted by C_GDM‐A_, performed well as predictor of GDM (accuracy = 72.6%; AUC = 0.720) during training (Figure 5; Table S11, Supporting Information). The internal cohort and two external validation cohorts for C_GDM‐A_ produces AUCs of 0.732 (0.663–0.800), 0.699 (0.604–0.794), and 0.711 (0.642–0.780), respectively (Figure 5; Table S8, Supporting Information). For PE, a set of ten genes, denoted by C_PE‐A_, performed well as predictor for PE (accuracy = 81.5% and AUC = 0.813) during training (Figure 5; Table S12, Supporting Information). The internal cohort and external validation cohorts for C_PE‐A_ produced AUCs of 0.898 (0.797–0.999) and 0.804 (0.710–0.899), respectively (Figure 5; Table S8, Supporting Information). All equations are shown in Table S13, Supporting Information.

### 2.4 Classifiers Based on Placenta‐ and Whole Blood‐Specific Genes

As the four complications examined in this study are associated with the dysfunctions of placenta and maternal immune system,^[^[qv: 28]^]^ placenta‐specific and blood‐specific genes may be better at predicting these complications. We thus developed three additional sets of classifiers: 1) set‐P classifiers, which were selected promoters of placenta‐specific genes; 2) set‐W classifiers, which were selected promoters of whole blood‐specific genes; and 3) set‐PW classifiers, which were selected promoters of placenta‐ and whole blood‐specific genes. Their procedures of classifier construction and validation were similar with those of set‐A classifiers. For whole blood‐specific genes, the optimal classifiers for MA (C_MA‐W_), FGR (C_FGR‐W_), GDM (C_GDM‐W_), and PE (C_PE‐W_) produced AUCs of 0.640 (0.591–0.689), 0.611 (0.563–0.658), 0.593 (0.559–0.627), and 0.69 (0.623–0.757), respectively (Figure 5; Table S13, Supporting Information). For placenta‐specific genes, the optimal classifiers for MA (C_MA‐P_), FGR (C_FGR‐P_), GDM (C_GDM‐P_), and PE (C_PE‐P_) produced AUCs of 0.644 (0.595–0.693), 0.616 (0.569–0.664), 0.618 (0.584–0.651), and 0.696 (0.63–0.762), respectively (Figure 5; Table S14, Supporting Information). For combination two types of tissue‐specific genes, the optimal classifiers for MA (C_MA‐PW_), FGR (C_FGR‐PW_), GDM (C_GDM‐PW_), and PE (C_PE‐PW_) produced AUCs of 0.649 (0.600–0.698), 0.621 (0.574–0.668), 0.626 (0.592–0.660), and 0.702 (0.636–0.768), respectively (Figure 5; Table S14, Supporting Information).

Although only approximately 10% of total cfDNA is derived from the placenta, with the rest majorly derived from maternal blood cells,^[^[qv: 27]^]^ the overall AUC of set‐P classifiers of four pregnancy complications was slightly higher than that of set‐W classifiers (Table S14, Supporting Information), indicating that placenta dysfunction may be one of the most factors underlying the occurrence of these four pregnancy complications, consistent with previous studies.^[^[qv: 28]^]^ We next compared the overall performance of the set‐A classifiers (derived from differential read depths of promoters without considering their tissues of origin) with those of the other three classifier sets for each pregnancy complication. Set‐A classifiers predicted complications more accurately than the other three classifier sets (Table S15, Supporting Information; all *p*‐value < 0.05), suggesting that factors other than dysfunctions of the placenta and maternal immune system may be related to the occurrence of these pregnancy complications.

### 2.5 Classifiers Combined with Clinical Features

Previous studies have reported that certain clinical features may be used to predict pregnancy complications, such as BMI before pregnancy. In our data, we found that their AUCs were significantly lower than set‐A classifiers for each complication (Table S16, Supporting Information; all *p*‐value < 0.05). To attempt to improve the performance of our classifiers, we further combined BMI with set‐A classifier of each complication (C_MA‐A_, C_FGR‐A_, C_GDM‐A_, and C_PE‐A_). But the AUC of the combined classifiers for predicting MA, GDM, and PE was decreased, whereas that of predicting FGR was slightly increased (Table S16, Supporting Information).

## 3 Discussion

In this study, we used promoter profiling of whole‐genome sequencing of cfDNA from pregnant women to assess the transcription activity of its tissues of origin. We found that differential read depths at the pTSS were indicative of differential gene expression in the tissues of origin, primarily the placenta and maternal blood (Figure 2). Therefore, we hypothesized that the differential read‐depth patterns of cfDNA at promoters should carry information regarding placenta‐origin diseases at an early stage, before any clinical symptoms become noticeable (Figure 1). To develop reliable predictors of subsequent pregnancy complications, we searched the genome for candidate promoters and implemented machine learning to select optimal classifiers for each pregnancy complication. Performance of the optimal classifiers for predicting MA (C_MA‐A_), FGR (C_FGR‐A_), GDM (C_GDM‐A_), and PE (C_PE‐A_) with an overall accuracy of 80.3%, 78.9%, 72.1%, and 83.0%, respectively (Table S8, Supporting Information). These findings highlight the potential predictive value of cfDNA read depth patterns as a non‐invasive assessment for predicting pregnancy complications at early gestational age.

The classifiers contained genes that may be correlated with the dysfunctions of pregnancy complications. For MA, the classifier C_MA‐A_ contained 12 genes *APC2*, *C1QTNF12*, *CREM*, *GPM6A*, *IGSF6*, *SMC3*, *MAP3K9*, *MASTL*, *MLXIP*, *NIPBL*, *TMEM128*, and *TMEM184A* (Table S9, Supporting Information). Previous studies have reported a close relationship between glucose metabolism and MA.^[^[qv: 30]^]^ Accordingly, *MLXIP* play a role in gene regulation in response to cellular glucose levels;^[^[qv: 31]^]^ *C1QTNF12* regulates glucose metabolism in liver and adipose tissues;^[^[qv: 32]^]^ and *CREM* is associated with Type 1 diabetes mellitus.^[^[qv: 33]^]^ For FGR, C_FGR‐A_ (Table S10, Supporting Information) included *CD63*, which is involved in different levels of platelet activation in preeclamptic, normotensive pregnant, and non‐pregnant women.^[^[qv: 34]^]^ For GDM, the classifier C_GDM‐A_ (Table S10, Supporting Information) included *CLOCK*, which may be associated with obesity and metabolic syndrome.^[^[qv: 35]^]^ For PE, the classifier C_PE‐A_ (Table S12, Supporting Information) contained *NFKB*, which is involved in inflammation and immune function, and associated with preterm birth.^[^[qv: 36]^]^ Further details regarding the function of individual genes in each classifier along with supporting citations can be found in Table S17, Supporting Information.

A useful biomarker for disease prediction requires low cost, easy detection and pervasive application. So far, no published studies have recruited more than 2000 pregnancies, began with high throughput screen, and performed validation in two independent external cohorts for predicting pregnancy complications. Our method was based on low‐coverage DNA sequencing data to predict pregnancy complications. The workflow of current noninvasive prenatal test (NIPT) procedures need not to change, therefore, our method can be easily adapted for preclinical tests based on current NIPT data. In addition, our method could simultaneously predict these four pregnancy complications based on the same sequencing data. With the accumulation of more NIPT data, this method might be applied to predict other severe pregnancy complications, such as preterm birth. Although the AUC, accuracy, sensitivity and specificity of our classifiers is robust in all cohorts, the positive prediction rate were approximately 0.5 (Table S18, Supporting Information). The low rate of positive prediction may come from the genetic heterogeneity of the diseases. For example, according to our recent study, the differentially expressed genes (DEGs) in clinical subtypes of PE are different: 2977 DEGs in early‐onset PE, 375 in late‐onset PE and 42 in late‐onset mild PE.^[^[qv: 37]^]^ The finer classification of the heterogeneous diseases may help improve the positive prediction. In addition, the performance of classifiers might be different among different ethnic groups. Previous studies have revealed that the risk factors of some pregnancy complications between different ethnic backgrounds were significantly different.^[^[qv: 38]^]^ In this study, we only validated the performance of our classifiers with one internal cohort and two external cohorts in Chinese. Therefore, such classifiers may be only applicable to Chinese patients.

The cfDNA and cfRNA have been taken as an important non‐invasive tool for the prediction of pregnancy complications. For cfDNA, it has been used to detect fetal chromosomal abnormalities in clinic,^[^[qv: 27]^]^ because detection of such abnormalities would not depend on the gene expression. In our study, we have developed classifiers using the promoter profiling of cfDNA for the prediction of pregnancy complications, based on the hypothesis that the promoter profiling of cfDNA may infer the gene expression patterns of maternal blood and placental tissues. The cfRNA can directly represent the expressed genes of maternal blood and placental tissues and thus may be used in the prediction of premature delivery.^[^[qv: 39]^]^

## 4 Conclusions

In summary, our data suggest that promoter‐profiling based classifiers provide high predictive capabilities for predicting multiple placenta‐origin pregnancy complications at early gestational age. The techniques required for low‐coverage DNA sequencing without additional tests, are easily applicable to routinely NIPT data, the results of classifiers are easy to interpret, and the costs of reagents and consumables are relatively low. Therefore, application of our classifiers in clinical practice should be feasible.

## 5 Experimental Section

##### Study Design and Participants

In this nested case‐control study, the authors developed classifiers for predicting pregnancy complication based on low coverage whole‐genome sequencing on the plasma cfDNA of 2199 participants. These participants included 578 pregnancies who developed MA (119 cases), FGR (132 cases), GDM (267 cases), and PE (60 cases) later on. In addition, 1621 controls were also sequenced, including controls for MA (476 cases), FGR (518 cases), GDM (801 cases), and PE (240 cases) (Table 1). Participants were enrolled at three independent hospitals of China, including Nanfang Hospital of Southern Medical University (SMU), The Third Affiliated Hospital of Sun Yat‐sen University (SYSU), and Cangzhou People's Hospital. The plasma used in the discovery and training cohort was collected between May 1, 2013, and Dec 31, 2016, at Nanfang Hospital. The samples used at the discovery stage were contained in the training cohort. The validation stage has three cohorts: 1) the internal validation cohort, enrolled at Nanfang Hospital from Jan 1, 2017, to Dec 31, 2017; 2) external validation cohort‐1, enrolled at The Third Affiliated Hospital from Jan 1, 2016, to Dec 31, 2017; 3) external validation cohort‐2 enrolled at Cangzhou People's Hospital from Jan 1, 2016 to Dec 31, 2017.

The DNA sequencing samples were selected from retrospective cohorts of 3200 participants according to their gestational age of plasma collection and follow‐up results. To develop classifiers for disease prediction, plasma samples had to be collected before the time period of each pregnancy complication diagnosis. All selected samples were collected at 12–28 weeks' gestation and the selected participants were singleton pregnancies. For MA and FGR, plasma was collected at 12–28 weeks' gestation. For GDM and PE, plasma samples were collected at <18 and <20 weeks' gestation, respectively. For healthy controls, their gestational age at the time of sample collection was matched with that of each pregnancy complication (Table S1, Supporting Information; *p* > 0.05, Mann–Whiney *U*‐test). According to the follow‐up results, we defined five groups of pregnancies including MA, FGR, GDM, PE, and healthy pregnancies. The detailed eligibility criteria of each group was listed in Supporting Information. Briefly, MA was defined as a birth weight ≥ 4000 g.^[^[qv: 40]^]^ FGR was defined as birth weight below the 10th percentile for gestational age^[^[qv: 41]^]^ and gestational age was ≥ 37 weeks. GDM was diagnosed according to International Association of Diabetes and Pregnancy Study Groups (IADPSG) criteria, with universal testing for GDM at 24–28 weeks' gestation with the 75 g 2 h OGTT. The PE definition was taken from the International Society for the Study of Hypertension in Pregnancy (ISSHP), which required blood pressure > 90 mmHg with proteinuria > 0.3 g in a 24 h collection > 20 weeks' gestation. Healthy control samples were collected from full‐term singleton pregnancies without pregnancy complications, in which the fetus was appropriately grown at birth with no obstetric, medical, or surgical complications in pregnancy. In addition, 300 healthy pregnancies in 1621 controls were selected to compare their promoter profiling with tissue expression profiles (Table S2, Supporting Information).

The institutional ethical review boards of all included hospitals approved this retrospective analysis and the requirement for informed consent was waived by the ethics review boards (NFEC‐2016‐093).

##### Sequence Analysis and Promoter Profiling

The procedure of DNA preparation, isolation and DNA sequencing are in the Supporting Information. Raw reads were aligned to the hg19 human reference genome using bwa‐mem,^[^[qv: 42]^]^ with PCR duplicates removed using the rmdup function of SAMtools (ver. 1.2).^[^[qv: 43]^]^ Gene information was obtained from the RefSeq of University of California Santa Cruz.^[^[qv: 44]^]^ For each transcript, the region ranging from −1 to +1 KB around the transcriptional start site defined as the primary transcription start site (pTSS) was identified. After alignment, read counts for each base at the pTSS were calculated from the aligned BAM files using SAMtools. The read coverage at the pTSS was extracted from the aligned BAM files using bedtools (ver. 2.17.0). The read counts were normalized using the reads per kilobase per million mapped reads (RPKM) method.

##### Microarray Data on the Placenta and Maternal Blood

Placenta and maternal blood expression profiles of healthy pregnancies (GSE85307 and GSE24129)^[^[qv: 45,46]^]^ and pregnancies with complications (GSE85307, GSE92772, GSE24129, and GSE70493)^[^[qv: 45–48]^]^ were downloaded from the GEO database. Normalized gene expression values were processed using GEO query in R (ver. 3.3.1). The 500 highest and lowest expressed genes in the placenta and maternal blood were identified (Table S3, Supporting Information). According to the methods adopted by previous studies,^[^[qv: 49]^]^ the Human 133A/GNF1H Gene Atlas Database (GSE1133)^[^[qv: 50]^]^ was analyzed using the MGFM package of R with default settings to identify placenta‐ and whole blood‐specific genes (Table S4, Supporting Information). The list of unexpressed genes in all tissues were downloaded from the Supporting Information of a previous study.^[^[qv: 26]^]^

##### Procedure of Classifiers Construction

At the discovery stage, we selected 10 cases of each pregnancy complication (MA, FGR, GDM, and PE) and 10 gestational age‐matched controls. Whole‐genome sequencing of cfDNA was then performed on each of these samples. Following alignment and normalization, promoter coverages at the pTSS were compared between each pregnancy complication and their corresponding controls, and a *p‐value* was calculated using the Wilcoxon rank sum test. *P‐*value was then adjusted to the false discovery rate (FDR) using the Benjamini–Hochberg procedure. Gene transcripts with FDR ≤ 0.1 and |log_2_ fold change| ≥ 1 were considered to have significant differential coverages at the pTSS (Table S6, Supporting Information).

At the training stage, we selected genes with significant differential coverages to develop promoter profiling‐based classifiers that could differentiate MA, FGR, GDM, and PE from healthy controls. The ten cases with complications and ten healthy controls used during the discovery stage were also included at the training stage. As a considerable amount of studies have reported that discrete data may improve classifier performance,^[^[qv: 51]^]^ the normalized read count of each promoter was discretized according to the optimal cut‐off point before classifier construction. The optimal cut‐off point of each promoter was defined as the maximum value of (sensitivity + specificity)/2 in the training cohort. The read depth of each promoter found in each subject was then set to one when it was larger than the corresponding optimal cut‐off; otherwise, it was set to zero. At the training stage, a stepwise method for feature selection was used to select the promoter combinations to construct classifiers. The robustness of these classifiers was assessed using the leave‐one‐out cross validation method. Briefly, each subject in the training cohort was withheld in turn, and the remaining subjects were submitted to train the model. The trained model was then used to predict the class (pregnancies with complications or healthy controls) of the withheld subject. This procedure went on until all subjects in the training cohort were judged. Receiver operating characteristic (ROC) analysis was used to evaluate the performance of each classifier, including area under curve (AUC), accuracy, sensitivity, and specificity. The classifiers which performed well and displayed the largest AUC in the training cohort were chosen as the optimal classifiers for each pregnancy complication (set‐A classifiers). The performance of these classifiers was then further validated using three independent validation cohorts, including one internal cohort and two external cohorts.

Apart from the set‐A classifiers (genes with significant differential coverages between pregnancy complication and healthy controls), another three sets of classifiers were developed to predict individual pregnancy complications: 1) set‐P classifiers selected promoters of placenta‐specific genes; 2) set‐W classifiers selected promoters of whole blood‐specific genes; and 3) set‐PW classifiers selected promoters of placenta‐ and whole blood‐specific genes. The procedure for selecting optimal classifiers for these sets was similar to that of the set‐A classifiers.

Previous studies have revealed that overweight and obesity were taken as significant contributors to disease.^[^[qv: 52]^]^ Therefore, pre‐pregnancy BMI potential for predicting pregnancy complications was also elevated. We first assessed BMI performance for predicting complications and compared its performance with set‐A classifiers. Then BMI was taken as a feature of set‐A classifiers to test whether the performance of combined classifiers would be increased.

##### Statistical Analyses

ROC curves and the significance of differences in the AUC, sensitivity and specificity were plotted and calculated using the pROC package in R.^[^[qv: 53]^]^ Maternal and gestational ages were compared between groups using the Mann–Whitney *U*‐test. The Wilcoxon rank sum test was used to identify genes with differential read coverages at the pTSS. χ^2^ test and Fisher exact test were used for comparison of categorical variables. *p*‐values < 0.05 for two sided tests were considered statistically significant. Hierarchical clustering was applied to the coverage data, using the average‐linkage clustering algorithms in Cluster (ver. 3.0). Heat maps were plotted using the pheatmap package in R. Function enrichment analysis was performed using Metascape with default setting.^[^[qv: 54]^]^ Then top ten enriched terms were visualized using ggplot2 and annotated by searching the literature.

## Conflict of Interest

The authors declare no conflict of interest.

## Supporting information

Supporting Information

## References

[1] B. Sibai , G. Dekker , M. Kupferminc , The Lancet 2005, 365, 785. 10.1016/S0140-6736(05)17987-2 15733721

[2] Y. Xu , L. Wang , J. He , Y. Bi , M. Li , T. Wang , Y. Jiang , M. Dai , J. Lu , M. Xu , Y. Li , N. Hu , J. Li , S. Mi , C. S. Chen , G. Li , Y. Mu , J. Zhao , L. Kong , J. Chen , S. Lai , W. Wang , W. Zhao , G. Ning , JAMA 2013, 310, 948.24002281

[3] L. Guariguata , D. R. Whiting , I. Hambleton , J. Beagley , U. Linnenkamp , J. E. Shaw , Diabetes Res. Clin. Pract. 2014, 103, 137. 24630390 10.1016/j.diabres.2013.11.002

[4] J. F. Froen , J. O. Gardosi , A. Thurmann , A. Francis , B. Stray‐Pedersen , Acta Obstet. Gynecol. Scand. 2004, 83, 801. 15315590 10.1111/j.0001-6349.2004.00602.x

[5] A. Mohammadbeigi , F. Farhadifar , N. Soufi Zadeh , N. Mohammadsalehi , M. Rezaiee , M. Aghaei , Ann. Med. Health Sci. Res. 2013, 3, 546. 24380006 10.4103/2141-9248.122098 PMC3868121

[6] C. A. Asplund , D. A. Seehusen , T. L. Callahan , C. Olsen , Ann. Fam. Med. 2008, 6, 550. 19001308 10.1370/afm.903 PMC2582460

[7] P. Damm , Int J Gynaecol Obstet 2009, 104, S25. 19150058 10.1016/j.ijgo.2008.11.025

[8] Y. M. Lo , T. N. Leung , M. S. Tein , I. L. Sargent , J. Zhang , T. K. Lau , C. J. Haines , C. W. Redman , Clin. Chem. 1999, 45, 184. 9931039

[9] D. Wright , D. M. Gallo , S. Gil Pugliese , C. Casanova , K. H. Nicolaides , Ultrasound Obstet. Gynecol. 2016, 47, 554. 26643929 10.1002/uog.15807

[10] Y. Zhong , F. Zhu , Y. Ding , BMC Pregnancy and Childbirth 2015, 15, 191. 26303460 10.1186/s12884-015-0608-y PMC4548561

[11] S. O. Diaz , J. Pinto , G. Graca , I. F. Duarte , A. S. Barros , E. Galhano , C. Pita , C. Almeida Mdo , B. J. Goodfellow , I. M. Carreira , A. M. Gil , J. Proteome Res. 2011, 10, 3732. 21649438 10.1021/pr200352m

[12] L. C. Kenny , D. I. Broadhurst , W. Dunn , M. Brown , R. A. North , L. McCowan , C. Roberts , G. J. Cooper , D. B. Kell , P. N. Baker , Hypertension 2010, 56, 741. 20837882 10.1161/HYPERTENSIONAHA.110.157297 PMC7614124

[13] R. O. Bahado‐Singh , R. Akolekar , R. Mandal , E. Dong , J. Xia , M. Kruger , D. S. Wishart , K. Nicolaides , Am. J. Obstet. Gynecol. 2013, 208, 58.e1. 23159745 10.1016/j.ajog.2012.11.003

[14] E. M. Tan , P. H. Schur , R. I. Carr , H. G. Kunkel , J. Clin. Invest. 1966, 45, 1732. 4959277 10.1172/JCI105479 PMC292857

[15] S. A. Leon , B. Shapiro , D. M. Sklaroff , M. J. Yaros , Cancer Res 1977, 37, 646. 837366

[16] J. Yi , Y. Zhang , Y. Ma , C. Zhang , Q. Li , B. Liu , Z. Liu , J. Liu , X. Zhang , R. Zhuang , B. Jin , Viruses 2014, 6, 2723. 25029493 10.3390/v6072723 PMC4113790

[17] J. Garnacho‐Montero , M. J. Huici‐Moreno , A. Gutierrez‐Pizarraya , I. Lopez , J. A. Marquez‐Vacaro , H. Macher , J. M. Guerrero , A. Puppo‐Moreno , Crit. Care 2014, 18, R116. 24903083 10.1186/cc13908 PMC4229882

[18] N. W. Tsai , T. K. Lin , S. D. Chen , W. N. Chang , H. C. Wang , T. M. Yang , Y. J. Lin , C. R. Jan , C. R. Huang , C. W. Liou , C. H. Lu , Clinica Chimica Acta 2011, 412, 476. 10.1016/j.cca.2010.11.036 21130757

[19] D. Tovbin , V. Novack , M. P. Wiessman , A. Abd Elkadir , M. Zlotnik , A. Douvdevani , Nephrol. Dial. Transplant. 2012, 27, 3929. 22833622 10.1093/ndt/gfs255

[20] C. Sur Chowdhury , S. Hahn , P. Hasler , I. Hoesli , O. Lapaire , S. Giaglis , Fetal Diagn. Ther. 2016, 40, 263. 26998969 10.1159/000444853

[21] T. Rafaeli‐Yehudai , M. Imterat , A. Douvdevani , D. Tirosh , N. Benshalom‐Tirosh , S. A. Mastrolia , R. Beer‐Weisel , V. Klaitman , R. Riff , S. Greenbaum , A. Alioshin , G. Rodavsky Hanegbi , G. Loverro , M. R. Catalano , O. Erez , PLoS One 2018, 13, e0200360. 30001403 10.1371/journal.pone.0200360 PMC6042756

[22] Y. M. Lo , K. C. Chan , H. Sun , E. Z. Chen , P. Jiang , F. M. Lun , Y. W. Zheng , T. Y. Leung , T. K. Lau , C. R. Cantor , R. W. Chiu , Sci. Transl. Med. 2010, 2, 61ra91. 10.1126/scitranslmed.3001720 21148127

[23] F. Diehl , M. Li , D. Dressman , Y. He , D. Shen , S. Szabo , L. A. Diaz Jr. , K. A. David , H. Juhl , K. W. Kinzler , B. Vogelstein , Proc. Natl. Acad. Sci. 2005, 102, 16368. 16258065 10.1073/pnas.0507904102 PMC1283450

[24] S. Ramachandran , S. Henikoff , Sci. Adv. 2015, 1, e1500587. 26269799 10.1126/sciadv.1500587 PMC4530793

[25] M. W. Snyder , M. Kircher , A. J. Hill , R. M. Daza , J. Shendure , Cell 2016, 164, 57. 26771485 10.1016/j.cell.2015.11.050 PMC4715266

[26] P. Ulz , G. G. Thallinger , M. Auer , R. Graf , K. Kashofer , S. W. Jahn , L. Abete , G. Pristauz , E. Petru , J. B. Geigl , E. Heitzer , M. R. Speicher , Nat. Genet. 2016, 48, 1273. 27571261 10.1038/ng.3648

[27] Y. M. Lo , M. S. Tein , T. K. Lau , C. J. Haines , T. N. Leung , P. M. Poon , J. S. Wainscoat , P. J. Johnson , A. M. Chang , N. M. Hjelm , Am. J. Hum. Genet. 1998, 62, 768. 9529358 10.1086/301800 PMC1377040

[28] J. Huynh , D. Dawson , D. Roberts , R. Bentley‐Lewis , Placenta 2015, 36, 101. 25524060 10.1016/j.placenta.2014.11.021 PMC4339292

[29] J. K. Park , J. W. Jeong , M. Y. Kang , J. C. Baek , J. K. Shin , S. A. Lee , W. S. Choi , J. H. Lee , W. Y. Paik , Placenta 2010, 31, 621. 20488538 10.1016/j.placenta.2010.04.009

[30] K. Kc , S. Shakya , H. Zhang , Ann Nutr Metab 2015, 2, 14. 10.1159/000371628 26045324

[31] P. Richards , L. Rachdi , M. Oshima , P. Marchetti , M. Bugliani , M. Armanet , C. Postic , S. Guilmeau , R. Scharfmann , Diabetes 2018, 67, 461. 29282201 10.2337/db17-0595

[32] T. Enomoto , K. Ohashi , R. Shibata , A. Higuchi , S. Maruyama , Y. Izumiya , K. Walsh , T. Murohara , N. Ouchi , J. Biol. Chem. 2011, 286, 34552. 21849507 10.1074/jbc.M111.277319 PMC3186379

[33] S. Nejentsev , L. J. Smink , D. Smyth , R. Bailey , C. E. Lowe , F. Payne , J. Masters , L. Godfrey , A. Lam , O. Burren , H. Stevens , S. Nutland , N. M. Walker , A. Smith , R. Twells , B. J. Barratt , C. Wright , L. French , Y. Chen , P. Deloukas , J. Rogers , I. Dunham , J. A. Todd , BMC Genetics 2007, 8, 24. 17509149 10.1186/1471-2156-8-24 PMC1885446

[34] M. R. Holthe , A. C. Staff , L. N. Berge , T. Lyberg , Am. J. Obstet. Gynecol. 2004, 190, 1128. 15118653 10.1016/j.ajog.2003.10.699

[35] S. Sookoian , C. Gemma , T. F. Gianotti , A. Burgueno , G. Castano , C. J. Pirola , Am. J. Clin. Nutr. 2008, 87, 1606. 18541547 10.1093/ajcn/87.6.1606

[36] D. R. Velez , S. J. Fortunato , P. Thorsen , S. J. Lombardi , S. M. Williams , R. Menon , PLoS One 2008, 3, e3283. 18818748 10.1371/journal.pone.0003283 PMC2553267

[37] Z Ren , Y Gao , G Liang , Q Chen , S Jiang , X Yang , C Fan , H Wang , J Wang , Yi‐Wu Shi , C Xiao , M Zhong , Y Yu , X Yang , bioRxiv 2019, 10.1101/787796.

[38] M. Hedderson , S. Ehrlich , S. Sridhar , J. Darbinian , S. Moore , A. Ferrara , Diabetes Care 2012, 35, 1492. 22619080 10.2337/dc11-2267 PMC3379591

[39] T. T. M. Ngo , M. N. Moufarrej , M. H. Rasmussen , J. Camunas‐Soler , W. Pan , J. Okamoto , N. F. Neff , K. Liu , R. J. Wong , K. Downes , R. Tibshirani , G. M. Shaw , L. Skotte , D. K. Stevenson , J. R. Biggio , M. A. Elovitz , M. Melbye , S. R. Quake , Science 2018, 360, 1133. 29880692 10.1126/science.aar3819 PMC7734383

[40] J. M. Walsh , F. M. McAuliffe , Eur. J. Obstet. Gynecol. Reprod. Biol. 2012, 162, 125. 22459652 10.1016/j.ejogrb.2012.03.005

[41] P. L. Yudkin , M. Aboualfa , J. A. Eyre , C. W. Redman , A. R. Wilkinson , Early Hum. Dev. 1987, 15, 45. 3816638 10.1016/0378-3782(87)90099-5

[42] B. Langmead , C. Trapnell , M. Pop , S. L. Salzberg , Genome Biol. 2009, 10, R25. 19261174 10.1186/gb-2009-10-3-r25 PMC2690996

[43] H. Li , B. Handsaker , A. Wysoker , T. Fennell , J. Ruan , N. Homer , G. Marth , G. Abecasis , R. Durbin , Bioinformatics 2009, 25, 2078. 19505943 10.1093/bioinformatics/btp352 PMC2723002

[44] J. Casper , A. S. Zweig , C. Villarreal , C. Tyner , M. L. Speir , K. R. Rosenbloom , B. J. Raney , C. M. Lee , B. T. Lee , D. Karolchik , A. S. Hinrichs , M. Haeussler , L. Guruvadoo , J. Navarro Gonzalez , D. Gibson , I. T. Fiddes , C. Eisenhart , M. Diekhans , H. Clawson , G. P. Barber , J. Armstrong , D. Haussler , R. M. Kuhn , W. J. Kent , Nucleic Acids Res. 2018, 46, D762. 29106570 10.1093/nar/gkx1020 PMC5753355

[45] H. Mirzakhani , A. A. Litonjua , T. F. McElrath , G. O'Connor , A. Lee‐Parritz , R. Iverson , G. Macones , R. C. Strunk , L. B. Bacharier , R. Zeiger , B. W. Hollis , D. E. Handy , A. Sharma , N. Laranjo , V. Carey , W. Qiu , M. Santolini , S. Liu , D. Chhabra , D. A. Enquobahrie , M. A. Williams , J. Loscalzo , S. T. Weiss , J. Clin. Invest. 2016, 126, 4702. 27841759 10.1172/JCI89031 PMC5127689

[46] H. Nishizawa , S. Ota , M. Suzuki , T. Kato , T. Sekiya , H. Kurahashi , Y. Udagawa , Reprod. Biol. Endocrinol. 2011, 9, 107. 21810232 10.1186/1477-7827-9-107 PMC3199758

[47] L. Stirm , P. Huypens , S. Sass , R. Batra , L. Fritsche , S. Brucker , H. Abele , A. M. Hennige , F. Theis , J. Beckers , M. Hrabe de Angelis , A. Fritsche , H. U. Haring , H. Staiger , Sci. Rep. 2018, 8, 1366. 29358694 10.1038/s41598-018-19200-9 PMC5778051

[48] A. M. Binder , J. LaRocca , C. Lesseur , C. J. Marsit , K. B. Michels , Clin. Epigenet. 2015, 7, 79. 10.1186/s13148-015-0116-y PMC4524439 26244062

[49] W. Koh , W. Pan , C. Gawad , H. C. Fan , G. A. Kerchner , T. Wyss‐Coray , Y. J. Blumenfeld , Y. Y. El‐Sayed , S. R. Quake , Proc. Natl. Acad. Sci. 2014, 111, 7361. 24799715 10.1073/pnas.1405528111 PMC4034220

[50] A. I. Su , T. Wiltshire , S. Batalov , H. Lapp , K. A. Ching , D. Block , J. Zhang , R. Soden , M. Hayakawa , G. Kreiman , M. P. Cooke , J. R. Walker , J. B. Hogenesch , Proc. Natl. Acad. Sci. 2004, 101, 6062. 15075390 10.1073/pnas.0400782101 PMC395923

[51] X. J. Lin , Y. Chong , Z. W. Guo , C. Xie , X. J. Yang , Q. Zhang , S. P. Li , Y. Xiong , Y. Yuan , J. Min , W. H. Jia , Y. Jie , M. S. Chen , M. X. Chen , J. H. Fang , C. Zeng , Y. Zhang , R. P. Guo , Y. Wu , G. Lin , L. Zheng , S. M. Zhuang , Lancet Oncol. 2015, 16, 804. 26088272 10.1016/S1470-2045(15)00048-0

[52] M. Ezzati , A. D. Lopez , A. Rodgers , S. Vander Hoorn , C. J. Murray , The Lancet 2002, 360, 1347. 10.1016/S0140-6736(02)11403-6 12423980

[53] X. Robin , N. Turck , A. Hainard , N. Tiberti , F. Lisacek , J. C. Sanchez , M. Muller , BMC Bioinformatics 2011, 12, 77. 21414208 10.1186/1471-2105-12-77 PMC3068975

[54] Y. Zhou , B. Zhou , L. Pache , M. Chang , A. H. Khodabakhshi , O. Tanaseichuk , C. Benner , S. K. Chanda , Nat. Commun. 2019, 10, 1523. 30944313 10.1038/s41467-019-09234-6 PMC6447622

