# Supplementary material for: Whole‐Genome Promoter Profiling of Plasma DNA Exhibits Diagnostic Value for Placenta‐Origin Pregnancy Complications
Source: Adv Sci (Weinh). 2020 Feb 18;7(7):1901819. doi: 10.1002/advs.201901819 (PMC7141029; doi:10.1002/advs.201901819)
Supplement: Supplementary file 1 — Supporting Information [file ADVS-7-1901819-s001.pdf]

Copyright WILEY-VCH Verlag GmbH & Co. KGaA, 69469 Weinheim, Germany, 2020.

Supporting Information

for *Adv. Sci.,* DOI: 10.1002/advs.201901819

Whole-Genome Promoter Profiling of Plasma DNA Exhibits Diagnostic Value for Placenta-Origin Pregnancy Complications

Zhiwei Guo, Fang Yang, Jun Zhang, Zhigang Zhang, Kun Li, Qi Tian, Hongying Hou, Cailing Xu, Qianwen Lu, Zhonglu Ren, Xiaoxue Yang, Zenglu Lv, Ke Wang, Xinping Yang, Yingsong Wu,* and Xuexi Yang**

Supporting Information

Whole-Genome Promoter Profiling of Plasma DNA Exhibits Diagnostic Value for Placenta-Origin Pregnancy Complications

Zhiwei Guo[1,†], *Fang Yang*[2,†], *Jun Zhang*[3,†], *Zhigang Zhang*[4,†], *Kun Li*[1], *Qi Tian*[3], *Hongying Hou*[3], *Cailing Xu*[2], *Qianwen Lu*[2], *Zhonglu Ren*[2], *Xiaoxue Yang*[2], *Zenglu Lv*[4], *Ke Wang*[2], *Xinping Yang*[2,*], *Yingsong Wu*[1,*], *Xuexi Yang*[1,*]

Supplemental methods

The definition of pregnancy complications and healthy controls

All selected samples for developing classifiers were singleton pregnancies.

Macrosomia was defined as a birth weight > 4,000-g[1] and gestational age at birth was $\geq$ 37 weeks.

Fetal growth restriction (FGR) was defined as a birth weight below the 10th percentile for gestational age[2] and gestational age at birth was $\geq$ 37 weeks.

Gestational diabetes mellitus (GDM) was defined according to International Association of Diabetes and Pregnancy Study Groups (IADPSG) criteria, with universal testing for GDM between 24 and 28 weeks' gestation with the 75-g 2-h OGTT. According to the IADPSG, at least one of the following criteria needs to be fulfilled for a GDM diagnosis: fasting plasma glucose, ≥ 5.1 mmol/L; 1-h glucose ≥ 10.0 mmol/L; 2-h glucose ≥ 8.5 mmol/L. In addition, pregnancies with diabetes mellitus type 1 or 2 were excluded.

Preeclampsia (PE) was defined according to the criteria of the International Society for the Study of Hypertension in Pregnancy (ISSHP), which required blood pressure ≥ 90 mmHg on at least two occasions, 4 h apart, with proteinuria ≥ 0.3-g in a 24-h collection after 20 weeks' gestation in previously normotensive women.

Participants due to chromosomal, congenital abnormalities, preterm birth and multiple pregnancies were excluded.

Healthy control samples were full-term singleton pregnancies without pregnancy complications where the fetus was appropriately grown at birth with no obstetric, medical, or surgical complications in pregnancy.

The number of control of case-control study

This is a nested case-control study. According to other case-control study published in journals with high impact factor,[3-5] the ratio of control and case is usually 3-5:1.

Base on the number of gestation age-matched healthy controls in our cohort, we selected four gestation age-matched healthy controls for MA, FGR and PE. As the number of gestation age-matched healthy controls was limited by 267 GDM cases, we selected three controls for each GDM case. The gestational age of four pregnancy complications and their corresponding controls were well matched in four cohorts (Supplemental Table S1; $p > 0.05$, Mann-Whiney *U* test). According to the time of sample collection and the sample size, the samples collected from Nanfang Hospital were divided into two cohorts, including training cohort (70% of samples) and internal cohort (30% of samples). The samples involved in The Third Affiliated Hospital of Sun Yat-sen University (SYSU) and Cangzhou People's Hospital were taken as external cohort-1 and external cohort-2, respectively. As some control samples were used in more than one pregnancy complication prediction, the number of total control is less than the sum control number for each pregnancy complication.

Isolation of cfDNA and whole-genome sequencing

Maternal blood was collected using a cfDNA BCT tube (Streck, USA). Cell-free plasma DNA (cfDNA) was extracted from a plasma sample using the QIAamp DNA Blood Mini kit (Qiagen, German) by following the manufacturer's instructions. DNA concentration and integration were measured using Qubit (ThermoFisher Scientific, USA) and Agilent Bioanalyzer 2100 (Agilent Technologies, USA). DNA was eluted in 50 μL AE buffer and stored at −20°C. For Life platform, libraries were prepared using the Ion Torrent Ampliseq 2.0 kit (ThermoFisher Scientific), according to the manufacturer's instructions. Samples were barcoded and quantified by qPCR using the Ion Xpress Barcode Adapter and the Ion Library TaqMan quantitation kit (ThermoFisher Scientific), respectively. Sequencing libraries were then sequenced on Ion Proton System on a P1 chip. For illumina platform, the DNA libraries were prepared using the TruSeq DNA Sample Prep reagents (Illumina, USA). After quantification on the LabChip GX microfluidic platform (Perkin- Elmer), the libraries were then sequenced using NextSeq. DNA sequencing was performed at a depth of 0.3× average coverage.

Reference:

[1] J. M. Walsh, F. M. McAuliffe, *Eur J Obstet Gynecol Reprod Biol* **2012**, *162*, 125.

[2] P. L. Yudkin, M. Aboualfa, J. A. Eyre, C. W. Redman, A. R. Wilkinson, *Early Hum Dev* **1987**, *15*, 45.

[3] D. A. Grimes, K. F. Schulz, *Lancet* **2005**, *365*, 1429.

[4] M. A. Hernan, A. J. Wilcox, *Epidemiology* **2009**, *20*, 167.

[5] Y. Lu, J. Lagergren, S. Eloranta, M. Lambe, *Epidemiology* **2009**, *20*, 780.

A list of Supplemental Figures and Supplemental Tables

ID	Description	File
Supplemental Figure 1	Promoter read depth patterns of highly and lowly expressed genes for pregnancy complications	In this file
Supplemental Figure 2	Functional enrichment analysis of genes with differential promoter coverages	In this file
Supplemental Table S1	Clinical characteristics of study pregnancies in four cohorts	In this file
Supplemental Table S2	Clinical characteristics of healthy pregnancies	In this file
Supplemental Table S3	500 most highest- and lowest-expressed genes in tissues	In a separated excel file
Supplemental Table S4	Tissue-specific genes of placenta and whole blood cells	In a separated excel file
Supplemental Table S5	The unexpressed genes in all tissues	In a separated excel file
Supplemental Table S6	Gene transcripts with differential read coverages at the pTSS	In a separated excel file
Supplemental Table S7	Functional annotation of genes with differential promoter coverage by retrieving literatures	In this file
Supplemental Table S8	The performance of classifiers for predicting pregnancy complications	In this file
Supplemental Table S9	The performance of the optimal gene combination in the training cohort for macrosomia	In this file
Supplemental Table S10	The performance of the optimal gene combination in the training cohort for FGR	In this file
Supplemental Table S11	The performance of the optimal gene combination in the training cohort for GDM	In this file
Supplemental Table S12	The performance of the optimal gene combination in the training cohort for PE	In this file
Supplemental Table S13	The logistical regression equations of classifiers	In this file
Supplemental Table S14	The performance of the optimal classifiers based on promoter profiling of tissue-specific genes	In this file

Supplemental Table S15	The AUC comparison between different classifier sets	In this file
Supplemental Table S16	The performance of clinical features for predicting pregnancy complications	In this file
Supplemental Table S17	Functional annotation of genes in classifiers by retrieving literatures	In this file
Supplemental Table S18	Performance evaluation of the classifiers	In this file

Supplemental Figures and legends

Supplemental Figures

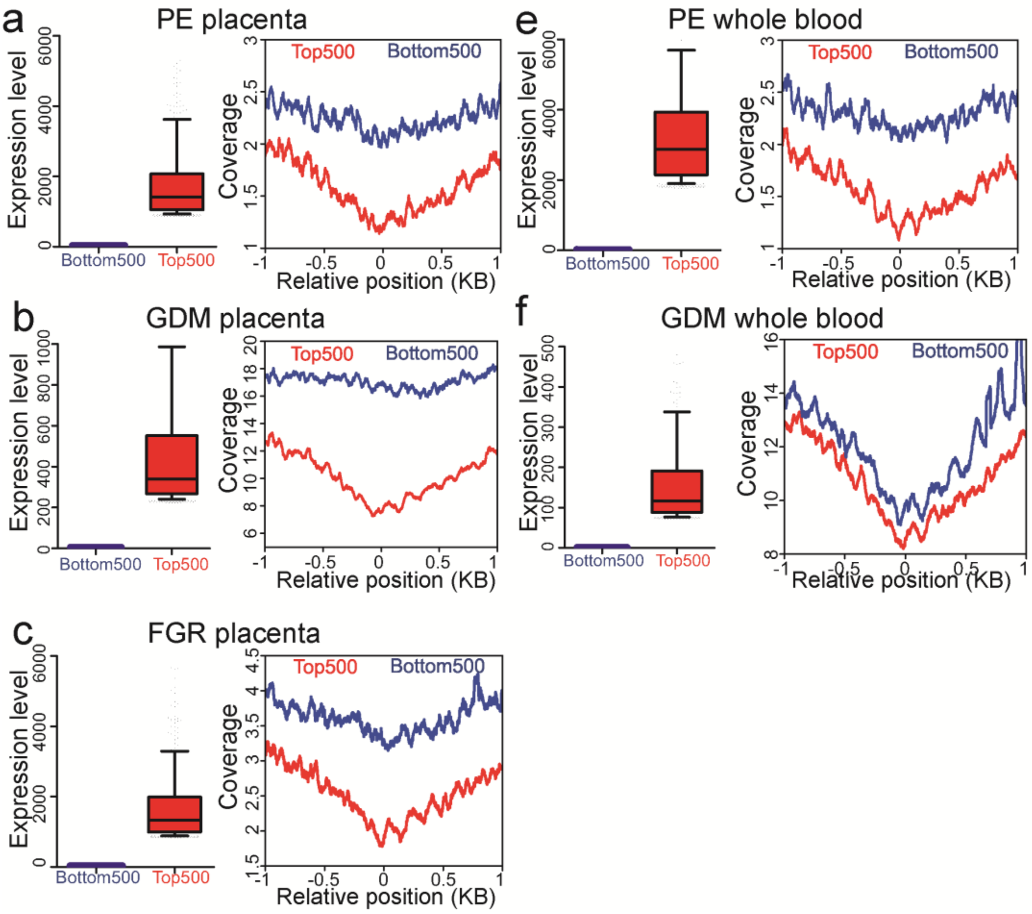

Supplemental Figure 1. Promoter read depth patterns of highly and lowly expressed genes for pregnancy complications. Mean expression levels of the 500 most- (Top500, red) and least-expressed (Bottom500, blue) genes in the placenta and their promoter read coverages of the cfDNA derived from pregnancies with PE **(a)**, GDM **(b)** and FGR **(c)**. Mean expression levels of the 500 most- and least-expressed genes in blood cells and their promoter read coverages of the cfDNA derived from pregnancies with PE **(d)** and GDM **(e)**. FGR = fetal growth restriction. GDM = gestational diabetes mellitus. PE = preeclampsia

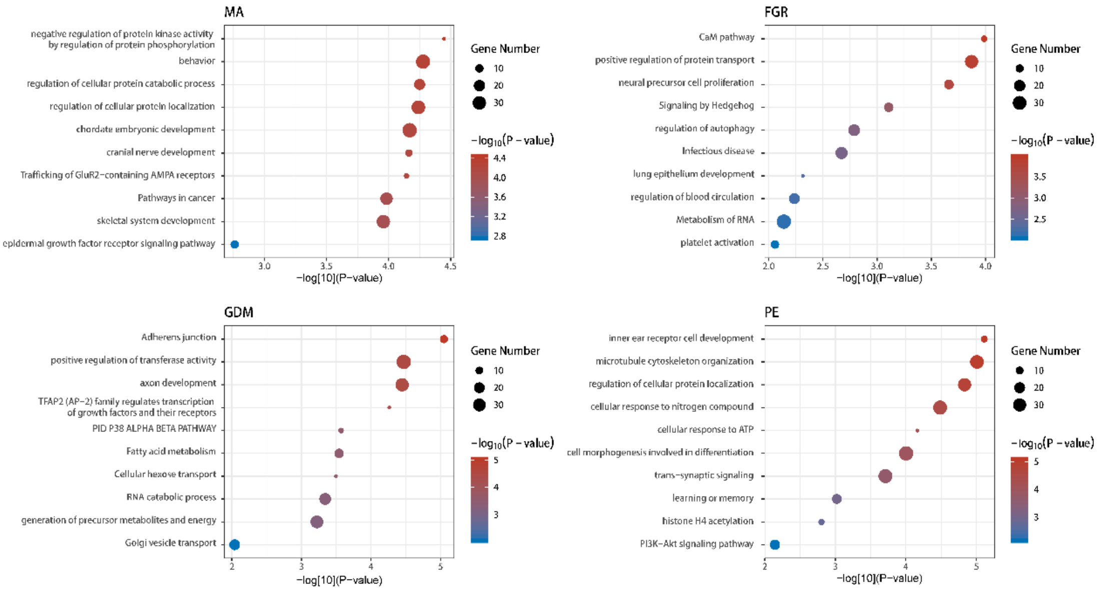

Supplemental Figure 2. Functional enrichment analysis of genes with differential promoter coverages. (a) MA. **(b)** FGR. **(c)** GDM. **(d)** PE. MA = macrosomia. FGR = fetal growth restriction. GDM = gestational diabetes mellitus. PE = preeclampsia

Supplementary Tables

Supplemental Table S1. Clinical characteristics of study pregnancies in four cohorts

		Gestational age at sampling					Maternal age			
		Training	Internal	External 1	External 2		Training	Internal	External 1	External 2
MA	Case	16.6+3.5	15.9+3	16.4+3.4	18.6+3.8		33.3+3.6	35.7+2.1	34.0+3.4	30.6+5.0
	Control	16.4+3.4	15.9+3.	16.4+3.3	18.8+3.9		31.7+5.0	32.1+5.1	34.0+3.3	30.1+4.9
	p-value	0.774	0.961	0.95	0.821		0.176	0.027	0.953	0.537
FGR	Case	19.3+5.	18.8+6.	19.4+6.2	18.3+3.9		30.9+5.	31.5+3.	31.3+5.2	31.0+6.1

		8	0				5	1		
	Control	18.2±4.8	18.4±5.1	19.2±5.7	18.3±3.9		31.8±4.9	31.9±4.6	31.4±5.2	30.5±5.3
	p-value	0.297	0.909	1.000	0.929		0.386	0.629	0.970	0.705
GDM	Case	15.2±1.8	15.2±1.9	16.7±1.3	16.3±1.6		34.5±4.0	34.5±3.5	32.5±5.3	33.6±5.4
	Control	15.3±2.0	15.2±2.0	16.7±1.3	16.3±1.3		32.9±5.1	32.7±4.4	32.1±5.1	33.3±3.8
	p-value	0.519	0.990	0.858	0.972		0.006	0.019	0.813	0.899
PE	Case	16.8±2.2	16.4±2.3	16.9±1.6	/		33.8±5.5	36.4±2.9	33.3±4.8	/
	Control	16.2±2.3	16.3±2.2	17.1±1.6	/		32.9±0.4	35.3±0.2	32.8±0.7	/
	p-value	0.903	0.925	0.659	/		0.357	0.188	0.664	/

Data are mean ± standard deviation. *p* = Mann-Whitney *U* test. MA = macrosomia. FGR = fetal growth restriction. GDM = gestational diabetes mellitus. PE = preeclampsia

Supplemental Table S2. Clinical characteristics of healthy pregnancies

	Range	Number (n=300)
Maternal age (years)	≤20	3
	21-25	29
	26-30	56
	31-35	139
	≥36	73
Gestational age at sampling (weeks)	≤12	23
	13-28	277

Supplemental Table S7. Functional annotation of genes with differential promoter coverage

by retrieving literatures

	Function	PMID
MA	Epidermal growth factor receptor signaling pathway	20817666<br>15592299
	Behavior	25256661
	Regulation of cellular protein localization	12646062
FGR	Infectious disease	12933859<br>18482615
	Metabolism of RNA	2581828
	Lung epithelium development	11536449
	Positive regulation of protein transport	19144403
	Regulation of blood circulation	11169323<br>11864679
	Regulation of autophagy	22815878
	Neural precursor cell proliferation	25720426
	Platelet activation	8193094
GDM	PID P38 ALPHA BETA PATHWAY	12937895<br>26508738
	Fatty acid metabolism	18849305<br>18986856
PE	Inner ear receptor cell development	20657326
	Microtubule cytoskeleton organization	15042020
	PI3K-Akt signaling pathway	20488538
	Regulation of cellular protein localization	12389018
	Histone H4 acetylation	31343798
	Learning or memory	28322777
	Cell morphogenesis involved in differentiation	23276825

PMID=PubMed Unique Identifier

Supplemental Table S8. The performance of classifiers for predicting pregnancy complications

	Performance			
	**AUC (95% CI)**	**Accuracy (%)**	**Sensitivity (%)**	**Specificity (%)**
**Training cohort**				
C_{MA-A}	0.766 (0.678-0.854)	80.0	71.0	82.3
C_{FGR-A}	0.774 (0.700-0.847)	79.5	73.8	81.0
C_{GDM-A}	0.720 (0.674-0.765)	72.6	70.6	73.3
C_{PE-A}	0.813 (0.727-0.898)	81.5	80.8	81.7
**Internal cohort**				
C_{MA-A}	0.817 (0.689-0.945)	84.6	76.9	86.5
C_{FGR-A}	0.813 (0.703-0.922)	81.3	81.3	81.3
C_{GDM-A}	0.732 (0.663-0.800)	72.7	74.1	72.2
C_{PE-A}	0.898 (0.797-0.999)	89.1	90.9	88.6
**External cohort-1**				
C_{MA-A}	0.791 (0.721-0.861)	80.5	76.7	81.4
C_{FGR-A}	0.763 (0.684-0.843)	79.5	71.1	81.6
C_{GDM-A}	0.699 (0.604-0.794)	71.0	67.7	72.0
C_{PE-A}	0.804 (0.710-0.899)	81.7	78.3	82.6
**External cohort-2**				
C_{MA-A}	0.762 (0.675-0.848)	78.8	71.9	80.5
C_{FGR-A}	0.765 (0.684-0.845)	76.5	76.5	76.5
C_{GDM-A}	0.711 (0.642-0.780)	71.0	71.4	70.8
C_{PE-A}	/	/	/	/
**All subjects**				
C_{MA-A}	0.779 (0.736-0.823)	80.3	73.9	81.9
C_{FGR-A}	0.773 (0.732-0.814)	78.9	74.6	80.0
C_{GDM-A}	0.718 (0.687-0.749)	72.1	71.2	72.4
C_{PE-A}	0.825 (0.770-0.880)	83.0	81.7	83.3

AUC = area under the receive operating characteristic curve. 95% CI = 95% confidence interval.

Supplemental Table S9. The performance of the optimal gene combination in the training cohort for macrosomia

	Gene	Cut-off†	Performances			
			AUC (95% CI)	Acc(%)	Sen(%)	Spe(%)
Individual gene in $C_{MA\text{-}A}$	SMC3	9.86	0.613(0.523-0.703)	53.6	74.2	48.4
	MASTL	7.41	0.621(0.523-0.719)	64.5	58.1	66.1
	CREM	9.54	0.653(0.560-0.747)	63.9	67.7	62.9
	C1QTNF12	7.45	0.633(0.538-0.729)	74.2	45.2	81.5
	MLXIP	7.41	0.577(0.480-0.673)	67.1	41.9	73.4
	MAP3K9	6.91	0.577(0.480-0.673)	67.1	41.9	73.4
	IGSF6	15.93	0.597(0.498-0.695)	64.5	51.6	67.7
	APC2	7.88	0.633(0.536-0.730)	70.3	51.6	75.0
	GPM6A	20.75	0.577(0.478-0.676)	59.4	54.8	60.5
	TMEM128	17.56	0.609(0.511-0.707)	64.5	45.2	62.9
	NIPBL	7.48	0.540(0.442-0.639)	59.4	54.8	37.1
	TMEM184A	11.49	0.637(0.540-0.734)	67.1	58.1	69.4
	12-genes combination	/	0.766(0.678-0.854)	80.0	71.0	82.3
Individual gene in $C_{MA\text{-}P}$	VAV3	16.71	0.625(0.527-0.723)	69.0	51.6	73.4
	COL4A1	17.20	0.633(0.536-0.73)	66.5	58.1	68.6
	LEPROT	9.84	0.569(0.471-0.666)	54.2	61.3	52.4
	PROCR	20.72	0.621(0.529-0.713)	56.8	71.0	53.2
	CRIM1	7.10	0.601(0.502-0.699)	65.2	51.6	68.6
	C5orf15	13.32	0.577(0.478-0.675)	57.4	58.1	57.3
	CRH	21.78	0.613(0.521-0.705)	55.5	71.0	51.6
	TMEM2	8.82	0.641(0.551-0.731)	58.1	74.2	54.0
	CTSL	18.85	0.573(0.474-0.672)	58.7	54.8	59.7
	9-genes combination	/	0.746(0.657-0.835)	76.8	71.0	78.2
Individual gene in $C_{MA\text{-}W}$	HSPA6	13.34	0.573(0.474-0.672)	60.7	51.6	62.9
	SYF2	14.56	0.589(0.494-0.683)	53.6	67.7	50.0
	CCPG1	12.88	0.561(0.461-0.660)	56.8	54.8	57.3
	PLEKHO2	7.08	0.561(0.461-0.660)	56.8	54.8	57.3
	TLE3	9.50	0.613(0.517-0.709)	59.4	64.5	58.1
	SUPT4H1	11.21	0.605(0.510-0.700)	56.1	67.7	53.2
	BID	13.19	0.609(0.514-0.703)	56.8	67.7	54.0
	CAB39	13.24	0.657(0.565-0.749)	62.6	71.0	60.5
	PF4V1	22.83	0.540(0.441-0.64)	53.6	54.8	53.2
	PBX2	9.49	0.552(0.453-0.652)	55.5	54.8	55.7
	MGAM	20.75	0.561(0.461-0.660)	58.7	51.6	60.5
	SDCBP	13.43	0.548(0.450-0.647)	52.9	58.1	51.6
	12-genes combination	/	0.718(0.628-0.808)	72.3	71.0	72.6

Individual gene in C_{MA-PW}	IFIT1	19.19	0.625(0.531-0.719)	59.4	67.7	57.3
	VAV3	16.71	0.625(0.527-0.723)	69.0	51.6	73.4
	HSPA6	13.34	0.573(0.474-0.672)	60.7	51.6	62.9
	COL4A1	17.20	0.633(0.536-0.73)	66.5	58.1	68.6
	SUSD6	9.25	0.613(0.518-0.707)	57.4	67.7	54.8
	TLE3	9.50	0.613(0.517-0.709)	59.4	64.5	58.1
	WFDC1	25.70	0.625(0.527-0.723)	69.0	51.6	73.4
	ARRB2	6.66	0.637(0.540-0.734)	67.1	58.1	69.4
	PROCR	20.72	0.621(0.529-0.713)	56.8	71.0	53.2
	MMP11	17.18	0.629(0.531-0.727)	67.7	54.8	71.0
	C5orf15	13.32	0.577(0.478-0.675)	57.4	58.1	57.3
	TMEM2	8.82	0.641(0.551-0.731)	58.1	74.2	54.0
	CTSL	18.85	0.573(0.474-0.672)	58.7	54.8	59.7
	13-genes combination	/	0.726(0.633-0.818)	77.4	64.5	80.7

†The optimal cut-off point of each gene was defined when the value of (sensitivity+specificity)/2 reached a maximum. AUC = area under the receive operating characteristic curve. 95% CI = 95% confidence interval. Acc = accuracy. Sen = sensitivity. Spe = specificity.

Supplemental Table S10. The performance of the optimal gene combination in the training cohort for FGR

	Gene	Cutoff†	Performances			
			AUC (95 CI)	Acc (%)	Sen (%)	Spe (%)
Individual gene in C_{FGR-A}	CD63	9.31	0.649(0.572-0.726)	59.5	73.8	56.0
	TCF7	12.08	0.604(0.524-0.684)	55.2	69.1	51.8
	OR4P4	10.06	0.592(0.512-0.674)	54.8	66.7	51.8
	VPS35	9.33	0.554(0.472-0.635)	48.6	66.7	44.1
	DNPEP	11.49	0.607(0.528-0.686)	54.3	71.4	50.0
	FAM214B	9.86	0.577(0.495-0.660)	53.8	64.3	51.2
	PTDSS2	7.20	0.598(0.514-0.682)	65.7	50.0	69.6
	GPAT4	13.26	0.607(0.524-0.691)	61.4	59.5	61.9
	LANCL2	16.26	0.577(0.493-0.662)	59.5	54.8	60.7
	VAV1	10.99	0.705(0.637-0.774)	62.9	83.3	57.7
	HPS5	10.53	0.622(0.539-0.705)	63.8	59.5	64.9
	DPP6	9.31	0.610(0.535-0.686)	51.9	76.2	45.8

	PNRC2	13.02	0.571(0.487-0.656)	55.7	59.5	54.8
	13-gene combination	/	0.774(0.700-0.847)	79.5	73.8	81.0
Individual gene in C_{FGR-P}	ADM	12.57	0.628(0.549-0.707)	57.6	71.4	54.2
	HSD17B1	15.52	0.622(0.540-0.704)	61.0	64.3	60.1
	MMP11	12.60	0.619(0.538-0.700)	59.0	66.7	57.1
	FBLN1	15.11	0.569(0.486-0.651)	52.4	64.3	49.4
	SLC5A6	14.06	0.625(0.550-0.700)	54.3	76.2	48.8
	CRIM1	9.86	0.589(0.509-0.670)	52.9	69	48.8
	ACVR2B	12.74	0.619(0.540-0.698)	56.2	71.4	52.4
	RYBP	7.62	0.542(0.460-0.624)	63.8	38.1	70.2
	PRKAG2	11.83	0.568(0.484-0.653)	55.2	59.5	54.2
	BZW2	11.02	0.613(0.531-0.695)	59.5	64.3	58.3
	AZIN1	6.74	0.521(0.438-0.603)	60.5	38.1	66.1
	LAMC3	17.83	0.679(0.605-0.752)	61.4	78.6	57.1
	12-genes combination	/	0.711 (0.633-0.790)	72.4	69.0	73.2
Individual gene in C_{FGR-W}	MAP3K11	9.19	0.536(0.451-0.621)	52.9	54.8	52.4
	PLXNC1	9.31	0.586(0.503-0.670)	56. 7	61.9	55.4
	CAP1	9.10	0.548(0.463-0.633)	54.8	54.8	54.8
	NUMB	9.33	0.563(0.478-0.648)	58.6	52.4	60.1
	MLH3	15.86	0.586(0.504-0.669)	55.2	64.3	53.0
	VMP1	17.08	0.566(0.481-0.650)	60.5	50.0	63.1
	ARPC2	9.32	0.631(0.550-0.712)	61.0	66.7	59.5
	PPP3R1	6.37	0.607(0.530-0.684)	52.9	73.8	47.6
	ARAP3	12.26	0.563(0.478-0.647)	57.1	54.8	57.7
	LST1	14.60	0.604(0.521-0.687)	59.5	61.9	58.9
	10-genes combination	/	0.670(0.588-0.751)	70.0	61.9	72.0
	ADM	12.57	0.628(0.549-0.707)	57.6	71.4	54.2
	CAP1	9.10	0.527(0.442-0.612)	51.4	54.8	50.6
	NUMB	9.33	0.586(0.504-0.669)	55.2	64.3	53.0
	MLH3	15.86	0.628(0.548-0.708)	59.1	69.1	56.6
	SERPINB2	24.34	0.598(0.515-0.681)	58.6	61.9	57.7
	ACVR2B	12.74	0.619(0.540-0.698)	56.2	71.4	52.4
	PRKAG2	11.83	0.569(0.484-0.653)	55.2	59.5	54.2
	PILRA	18.10	0.563(0.478-0.647)	57.1	54.8	57.7
	FAM49B	13.48	0.604(0.521-0.687)	59.5	61.9	58.9
	10-genes combination	/	0.688 (0.608-0.768)	70.0	66.7	70.8

†The optimal cut-off point of each gene was defined when the value of (sensitivity+specificity)/2 reached a maximum. AUC = area under the receive operating characteristic curve. 95% CI = 95% confidence interval. Acc = accuracy. Sen = sensitivity. Spe = specificity.

Supplemental Table S11. The performance of the optimal gene combination in the training cohort for GDM

	Gene	Cutoff†	Performances			
			AUC (95% CI)	Acc(%)	Sen(%)	Spe(%)
Individual gene in C_{GDM-A}	CC2D2B	18.38	0.579(0.529-0.629)	61.1	51.6	64.3
	NAT10	16.96	0.616(0.570-0.663)	56.8	71.4	51.9
	SIPA1	16.83	0.614(0.568-0.660)	54.8	74.6	48.2
	ZNF565	13.92	0.583(0.537-0.630)	51.0	73.0	43.7
	ZNF552	16.80	0.582(0.536-0.628)	50.4	73.8	42.6
	WDR35	17.97	0.549(0.500-0.600)	50.6	63.5	46.3
	MICALL1	10.68	0.583(0.533-0.633)	60.5	54.0	62.7
	CTNNB1	13.73	0.573(0.525-0.620)	51.4	69.1	45.5
	CLOCK	13.93	0.575(0.527-0.624)	53.0	66.7	48.4
	BCKDHB	14.44	0.573(0.523-0.623)	56.2	59.5	55.0
	TGIF2LY	7.26	0.593(0.544-0.642)	56.8	64.3	54.2
	11-genes combination	/	0.720(0.674-0.765)	72.6	70.6	73.3
Individual gene in C_{GDM-P}	RBMS2	14.89	0.561(0.513-0.609)	50.0	31.8	56.1
	IFI6	20.20	0.564(0.513-0.614)	57.1	54.8	57.9
	HSD17B2	22.41	0.579(0.529-0.629)	59.1	55.6	60.3
	DLX4	17.42	0.565(0.515-0.615)	55.4	58.7	54.2
	CSHL1	20.22	0.550(0.500-0.600)	53.2	58.7	51.3
	LGALS14	23.77	0.564(0.514-0.613)	54.0	61.1	51.6
	EPS8L1	15.41	0.554(0.504-0.604)	53.8	58.7	52.1
	PROCR	20.10	0.546(0.496-0.597)	56.9	50.0	59.3
	DAG1	9.64	0.554(0.504-0.604)	54.6	57.1	53.7
	SQSTM1	7.70	0.534(0.484-0.585)	55.2	50.0	56.9
	AZIN1	7.91	0.515(0.464-0.565)	52.2	50.0	52.9
	CTSL	16.61	0.586(0.538-0.634)	54.6	67.0	50.5
	12-genes combination	/	0.652(0.604-0.700)	66.5	62.7	67.7

Individual gene in C_{GDM-W}	CREBBP	7.26	0.519(0.468-0.569)	52.0	51.6	52.1
	SUPT4H1	10.57	0.566(0.518-0.615)	51.6	66.7	46.6
	CTBS	15.23	0.593(0.543-0.642)	57.5	62.7	55.8
	ARPC2	8.37	0.534(0.484-0.585)	55.2	50.0	56.9
	ASXL2	10.98	0.523(0.472-0.573)	51.0	54.8	49.7
	PF4	18.60	0.545(0.495-0.595)	56.8	50.0	59.0
	HIST1H2BC	10.98	0.570(0.520-0.620)	55.8	59.5	54.5
	SDCBP	10.69	0.558 (0.51-0.606)	50.0	67.5	44.2
	TOPORS	14.83	0.562(0.513-0.611)	52.6	63.5	48.9
	9-genes combination	/	0.626(0.576-0.675)	64.5	58.7	66.4
Individual gene in C_{GDM-PW}	FRAT1	6.94	0.554(0.504-0.605)	57.3	51.6	59.3
	IFI6	20.20	0.564(0.513-0.614)	57.1	54.8	57.9
	HSD11B2	11.15	0.523(0.472-0.573)	52.6	51.6	52.9
	HSD17B2	22.41	0.579(0.529-0.629)	59.1	55.6	60.3
	DLX4	17.42	0.565(0.515-0.615)	55.4	58.7	54.2
	CSHL1	20.22	0.550(0.500-0.600)	53.2	58.7	51.3
	LGALS14	23.77	0.564(0.514-0.613)	53.9	61.1	51.6
	ARPC2	8.37	0.534(0.484-0.585)	55.2	50.0	56.9
	HIST1H2BC	10.98	0.570(0.520-0.620)	55.8	59.5	54.5
	TMEM2	10.27	0.550(0.500-0.600)	54.8	55.6	54.5
	VGLL1	23.50	0.537(0.487-0.588)	53.6	54.0	53.4
	11-genes combination	/	0.647(0.598-0.696)	67.3	59.5	69.8

[†]The optimal cut-off point of each gene was defined when the value of (sensitivity+specificity)/2 reached a maximum. AUC = area under the receive operating characteristic curve. 95% CI = 95% confidence interval. Acc = accuracy. Sen = sensitivity. Spe = specificity.

Supplementary Table S12. The performance of the optimal gene combination in the training cohort for PE

	Gene	Cutoff†	Performances			
			AUC (95% CI)	Acc(%)	Sen(%)	Spe(%)
Individual gene in C_{PE-A}	NFKB2	8.37	0.625(0.520-0.730)	60.8	65.4	59.6
	EHBP1L1	9.36	0.639(0.540-0.739)	58.5	73.1	54.8
	AMOTL1	13.10	0.606(0.498-0.713)	62.3	57.7	63.5
	VSIG10	12.19	0.615(0.509-0.722)	61.5	61.5	61.5
	USP10	9.75	0.577(0.469-0.685)	60.0	53.9	61.5
	ZSWIM4	6.26	0.582(0.476-0.687)	67.7	42.3	74.0
	ZNF565	13.17	0.625(0.519-0.731)	63.1	61.5	63.5
	BZW1	8.31	0.625(0.520-0.730)	60.8	65.4	59.6
	ATP6V1E2	10.58	0.606(0.499-0.712)	60.0	61.5	59.6
	CDX1	22.03	0.649(0.544-0.755)	66.9	61.5	68.3
	10-genes combination	/	0.813(0.727-0.898)	81.5	80.8	81.7
Individual gene in C_{PE-P}	ITIH5	12.39	0.6394(0.54-0.739)	58.5	73.1	54.8
	ST3GAL4	13.38	0.606(0.498-0.713)	62.3	57.7	63.5
	EVA1B	16.40	0.615(0.509-0.722)	61.5	61.5	61.5
	PSG1	11.12	0.582(0.476-0.687)	67.7	42.3	74
	PSG7	24.95	0.635(0.529-0.741)	64.6	61.5	65.4
	SDC1	11.15	0.625(0.519-0.731)	63.1	61.5	63.5
	AGTR1	29.67	0.707(0.616-0.797)	64.6	80.8	60.6
	RYBP	8.32	0.615(0.511-0.72)	59.2	65.4	57.7
	AFF1	14.89	0.625(0.520-0.73)	60.8	65.4	59.6
	TNS3	15.16	0.606(0.499-0.712)	60.0	61.5	59.6
	GNE	12.46	0.659(0.573-0.7442)	54.6	84.6	47.1
	HMGB3	11.59	0.649(0.544-0.755)	66.9	61.5	68.3
	12-genes combination	/	0.736(0.639-0.832)	73.85	73.08	74.04
Individual gene in C_{PE-W}	PXN	14.45	0.611(0.503-0.718)	67.7	50.0	72.1
	CAP1	8.14	0.596(0.490-0.703)	58.5	61.5	57.7
	MLH3	13.57	0.51(0.400-0.619)	51.5	50.0	51.9
	CCPG1	16.09	0.558(0.449-0.666)	56.9	53.8	57.7
	CRLF3	10.80	0.611(0.504-0.717)	60.8	61.5	60.6
	VMP1	16.70	0.563(0.456-0.669)	53.1	61.5	51.0
	CTBS	17.31	0.591(0.484-0.699)	60.0	57.7	60.6
	VASP	12.21	0.572(0.470-0.675)	50.0	69.2	45.2
	CAB39	6.63	0.644 (0.539-0.75)	73.1	50.0	78.8

	PPP3R1	9.46	0.625(0.520-0.730)	60.8	65.4	59.6
	13-genes combination	/	0.683(0.580-0.786)	70.0	65.4	71.2
Individual gene in C_{PE-PW}	ITIH5	12.39	0.673(0.574-0.772)	63.9	73.1	61.5
	PXN	14.45	0.611(0.503-0.718)	67.7	50.0	72.1
	CAP1	8.14	0.596(0.49-0.703)	58.5	61.5	57.7
	EFS	10.64	0.625(0.518-0.732)	65.4	57.7	67.3
	CCPG1	16.09	0.558(0.449-0.666)	56.9	53.8	57.7
	PSG1	11.12	0.639(0.535-0.744)	63.1	65.4	62.5
	VASP	12.21	0.572(0.470-0.675)	50.0	30.8	54.8
	PPP3R1	9.46	0.625(0.520-0.730)	60.8	65.4	59.6
	ABHD5	9.36	0.591(0.507-0.676)	56.2	15.4	66.4
	RYBP	8.32	0.615(0.508-0.723)	66.2	53.9	69.2
	IRF2	6.61	0.606(0.499-0.713)	66.9	50.0	71.2
	TNS3	15.16	0.611(0.503-0.718)	63.1	57.7	64.4
	ZCCHC6	12.81	0.601(0.498-0.704)	54.6	69.2	51.0
	13-genes combination	/	0.702(0.599-0.805)	75.4	61.5	78.9

[†]The optimal cut-off point of each gene was defined when the value of (sensitivity+specificity)/2 reached a maximum. AUC = area under the receive operating characteristic curve. 95% CI = 95% confidence interval. Acc = accuracy. Sen = sensitivity. Spe = specificity.

Supplemental Table S13. The logistical regression equations of classifiers

**Classifier**	**Equation (Logit [p=pregnancies with MA/FGR/GDM/PE])**	**Threshold**
C_{MA-A}	C_{MA-A}= 2.180 + 0.605 × SMC3 – 1.204 × MASTL + 1.366 × CREM – 1.295 × C1QTNF12 – 0.471 × MLXIP – 0.811 × MAP3K9 – 1.284 × IGSF6 – 1.347 × APC2 – 0.504 × GPM6A + 1.048 × TMEM128 – 0.057 × NIPBL – 1.652 × TMEM184A	0.277
C_{MA-P}	C_{MA-P}= – 2.007 + 1.269 ×VAV3 + 1.405 × COL4A1 + 0.240 × LEPROT – 1.221 × PROCR – 0.893 × CRIM1 – 0.926 × C5orf15 – 1.198 × CRH + 1.201 × TMEM2 + 1.213 × CTSL	0.220
C_{MA-W}	C_{MA-W}= – 2.556 – 1.003 × HSPA6 + 1.136 × SYF2 + 0.602 × CCPG1 – 0.1503 × PLEKHO2 + 0.887 × TLE3 + 1.224 × SUPT4H1 – 1.528 × BID + 1.879 × CAB39 – 0.208 × PF4V1 + 0.402 × PBX2 – 1.011 × MGAM – 0.597 × SDCBP	0.188
C_{MA-PW}	C_{MA-PW} = – 4.382 + 0.958 × IFIT1 + 1.035 × VAV3 – 0.982 × HSPA6 + 1.251 × COL4A1 + 1.461 × SUSD6 + 0.471 × TLE3 – 0.845 × ARRB2 + 1.266 × MMP11 + 1.457 × CAB39 – 1.064 × CRIM1 – 1.379 × C5orf15 + 1.175 × TMEM2 + 1.168 ×	0.250

	CTSL	
C_{FGR-A}	C_{FGR-A}= – 1 – 0.797 × HPS5 – 1.142 × PTDSS2 + 0.846 × OR4P4 + 0.636 × PNRC2 + 1.208 × CD63 + 0.310 × VPS35 – 1.903 × VAV1 – 1.313 × DNPEP – 1.370 × TCF7 + 0.673 × DPP6 + 0.574 × LANCL2 – 1.288 × GPAT4 + 1.238 × FAM214B	0.190
C_{FGR-P}	C_{FGR-P}= 0.360 – 1.489 × ADM – 0.704 × HSD17B1 – 0.969 × MMP11 – 0.345 × FBLN1 – 1.229 × SLC5A6 + 1.218 × CRIM1 – 1.079 × ACVR2B – 0.389 × RYBP – 0.273 × PRKAG2 – 0.961 × BZW2 – 0.154 × AZIN1 + 1.402 × LAMC3	0.190
C_{FGR-W}	C_{FGR-W}= – 2.393 – 0.538 × MAP3K11 + 0.5826 × PLXNC1 + 0.23 × CAP1 + 0.619 × NUMB + 0.849 × MLH3 + 0.764 × VMP1 – 1.209 × ARPC2 + 0.949 × PPP3R1 + 0.480 × ARAP3 –1.138 × LST1	0.222
C_{FGR-PW}	C_{FGR-PW}= – 1.867 – 1.154 × ADM + 0.441 × CAP1 + 0.700 × NUMB + 1.166 × MLH3 + 0.852 × SERPINB –1.105 × ACVR2B – 0.211× PRKAG2+0.545 × PILRA – 1.030 × FAM49B	0.208
C_{GDM-A}	C_{GDM-A}= 0.957 + 0.565 × CC2D2B – 1.060 × NAT10 – 1.070 × SIPA1 – 0.620 × ZNF565 – 0.805 × ZNF552 – 0.367 × WDR35 + 0.559 × MICALL1 – 0.653 × CTNNB1 – 0.529 × CLOCK –0.674 × BCKDHB – 0.693 × TGIF2LY	0.259
C_{GDM-P}	C_{GDM-P}= – 0.886 + 0.699 × RBMS2 – 0.529 × IFI6 – 0.867 × HSD17B2 – 0.496 × DLX4 – 0.701 × CSHL1 – 0.676 × LGALS14 + 0.469 × EPS8L1 + 0.439 × PROCR + 0.587 × DAG1 – 0.350 × SQSTM1 – 0.179 × AZIN1 + 0.812 × CTSL	0.251
C_{GDM-W}	C_{GDM-W}= –1.82265 – 0.073 × CREBBP + 0.595 × SUPT4H1 + 0.728 × CTBS – 0.202 × ARPC2 + 0.145 × ASXL2 – 0.403 × PF4 + 0.544 × HIST1H2BC + 0.398 × SDCBP – 0.626 × TOPORS	0.261
C_{GDM-PW}	C_{GDM-PW}=–0.217 – 0.456 × FRAT1 – 0.489 × IFI6 + 0.159 × HSD11B2 – 0.713 × HSD17B2 – 0.617 × DLX4 – 0.568 × CSHL1 – 0.563 × LGALS14 – 0.115 × ARPC2 + 0.662 × HIST1H2BC + 0.407 × TMEM2 + 0.469 × VGLL1	0.263
C_{PE-A}	C_{PE-A}= –0.655 – 1.146 × NFKB2 + 1.350 × EHBP1L1 – 1.371 × AMOTL1 – 0.784 × VSIG10 –1.047 × USP10 – 1.226 × ZSWIM4 + 1.242 × ZNF565 – 0.983 × BZW1 + 0.761 × ATP6V1E2 + 1.842 × CDX1	0.258
C_{PE-P}	C_{PE-P}= –1.745 + 1.500 × ITIH5 – 0.524 × ST3GAL4 – 0.301 × EVA1B – 1.22 × PSG1 + 1.056 × PSG7 –1.267 × SDC1 – 1.45 × AGTR1 – 1.065 × RYBP + 0.445 × AFF1 + 1.659 × TNS3 –0.163 × GNE +1.273 × HMGB3	0.170
C_{PE-W}	C_{PE-W}= –1.756 + 1.224 × PXN – 0.480 × CAP1 – 0.010 × MLH3 + 0.140 × CCPG1 + 0.786 × CRLF3 – 0.605 × VMP1 + 0.662 × CTBS – 1.021 × VASP – 1.228 × CAB39 + 1.536 × PPP3R1	0.201
C_{PE-PW}	C_{PE-PW}= –1.634 + 1.546 × ITIH5 + 0.825 × PXN – 0.656 × CAP1 – 0.984 × EFS + 0.358 × CCPG1 – 1.066 × PSG1 – 0.658 × VASP + 1.379 × PPP3R1 + 0.531 × ABHD5 – 0.461 × RYBP – 0.973 × IRF2 + 0.967 × TNS3 – 0.440 × ZCCHC6	0.216

In these equations, the gene coverage around TSSs ranging from –1 KB to +1 KB were substituted with the discretized value “one” when the level of each gene was larger than the corresponding cut-off (see *Supplemental Table S8-S11*); otherwise, it was substituted with the discretized value “zero”. If the result of Logit p was larger than the corresponding threshold, the detected subject was predicted as pregnancy complications (MA, GFR, GDM, and PE); otherwise as non-obstetrical syndromes. MA = macrosomia. FGR = fetal growth restriction. GDM = gestational diabetes mellitus. PE = preeclampsia.

Supplemental Table S14. The performance of the optimal classifiers based on promoter profiling of tissue-specific genes

	Whole blood-specific genes					Placenta-specific genes					Whole blood-and placenta-specific genes			
	AUC (95% CI)	ACC	Sen	Spe		AUC (95% CI)	ACC	Sen	Spe		AUC (95% CI)	ACC	Sen	Spe
**Training cohort**														
C_{MA-W}	0.718(0.628-0.808)	72.3	71.0	72.6	C_{MA-P}	0.746(0.657-0.835)	76.8	71.0	78.2	C_{MA-PW}	0.766 (0.678-0.854)	80.0	71.0	82.3
C_{FGR-W}	0.670(0.588-0.751)	70.0	61.9	72.0	C_{FGR-P}	0.711(0.633-0.790)	72.4	69.0	73.2	C_{FGR-PW}	0.688(0.608-0.768)	70.0	66.7	70.8
C_{GDM-W}	0.626(0.576-0.675)	64.5	58.7	66.4	C_{GDM-P}	0.652(0.604-0.701)	66.5	62.7	67.7	C_{GDM-PW}	0.647(0.598-0.696)	67.3	59.5	69.8
C_{PE-W}	0.683(0.580-0.786)	70.0	65.4	71.2	C_{PE-P}	0.736(0.639-0.832)	73.8	73.1	74.0	C_{PE-PW}	0.702(0.599-0.805)	75.4	61.5	78.8
**Internal cohort**														
C_{MA-W}	0.625(0.472-0.778)	63.1	61.5	63.5	C_{MA-P}	0.644(0.492-0.796)	66.2	61.5	67.3	C_{MA-PW}	0.635(0.482-0.787)	64.6	61.5	65.4
C_{FGR-W}	0.586(0.447-0.725)	63.8	50.0	67.2	C_{FGR-P}	0.570(0.431-0.710)	61.3	50.0	64.1	C_{FGR-PW}	0.625(0.487-0.763)	66.3	56.3	68.8
C_{GDM-W}	0.562(0.485-0.639)	59.3	50.0	62.3	C_{GDM-P}	0.679(0.606-0.753)	70.4	63.0	72.8	C_{GDM-PW}	0.633(0.557-0.708)	65.3	59.3	67.3
C_{PE-W}	0.739(0.586-0.891)	74.5	72.7	75.0	C_{PE-P}	0.750(0.598-0.902)	76.4	72.7	77.3	C_{PE-PW}	0.750(0.598-0.902)	76.4	72.7	77.3
**External cohort-1**														
C_{MA-W}	0.619(0.537-0.702)	64.2	58.1	65.7	C_{MA-P}	0.663(0.582-0.744)	69.8	60.5	72.1	C_{MA-PW}	0.640(0.557-0.722)	68.8	55.8	72.1
C_{FGR-W}	0.602(0.514-0.690)	66.3	50.0	70.4	C_{FGR-P}	0.635(0.548-0.722)	71.6	50.0	77.0	C_{FGR-PW}	0.625(0.537-0.713)	66.8	55.3	69.7
C_{GDM-W}	0.610(0.535-0.685)	64.7	53.6	68.5	C_{GDM-P}	0.554(0.478-0.629)	58.0	50.0	60.7	C_{GDM-PW}	0.616(0.542-0.690)	62.1	60.7	62.5
C_{PE-W}	0.674(0.562-0.785)	71.3	60. 9	73.9	C_{PE-P}	0.625(0.512-0.738)	63.5	60.9	64.1	C_{PE-PW}	0.679(0.572-0.787)	67.0	69.6	66.3
**External cohort-2**														
C_{MA-W}	0.598(0.501-0.694)	65.6	50.0	69.5	C_{MA-P}	0.520(0.422-0.617)	55.0	46.9	57.0	C_{MA-PW}	0.555(0.457-0.652)	60.6	46.9	64.1
C_{FGR-W}	0.559(0.465-0.653)	54.1	58.8	52.9	C_{FGR-P}	0.500(0.416-0.584)	64.1	26.5	73.5	C_{FGR-PW}	0.533(0.441-0.625)	62.4	38.2	68.4
C_{GDM-W}	0.484(0.382-0.586)	51.6	41.9	54.8	C_{GDM-P}	0.489(0.387-0.591)	52.4	41.9	55.9	C_{GDM-PW}	0.5484(0.446-0.651)	58.1	48.4	61.3
C_{PE-W}	/	/	/	/	C_{PE-P}	/	/	/	/	C_{PE-PW}	/	/	/	/
**All subjects**														
C_{MA-W}	0.640(0.591-0.689)	66.6	59.7	68.3	C_{MA-P}	0.644(0.595-0.693)	67.2	59.7	69.1	C_{MA-PW}	0.649 (0.600-0.698)	69.1	58.0	71.8
C_{FGR-W}	0.611(0.563-0.658)	64.0	56.2	66.0	C_{FGR-P}	0.616(0.569-0.664)	68.6	50.0	73.3	C_{FGR-PW}	0.621 (0.574-0.668)	66.7	54.6	69.6
C_{GDM-W}	0.593(0.559-0.627)	62.0	53.9	64.7	C_{GDM-P}	0.618(0.584-0.652)	63.9	57.7	65.9	C_{GDM-PW}	0.626 (0.592-0.660)	64.7	58.4	66.8
C_{PE-W}	0.690(0.623-0.757)	71.3	65.0	72.9	C_{PE-P}	0.696(0.630-0.762)	70.3	68.3	70.8	C_{PE-PW}	0.702 (0.636-0.768)	72.3	66.7	73.8

AUC = area under the receive operating characteristic curve. 95% CI = 95% confidence interval. Acc = accuracy. Sen = sensitivity. Spe = specificity.

Supplemental Table S15. The AUC comparison between different classifier sets

	**AUC (95% CI)**	**p-value**
MA		
C_{MA-A}	0.779 (0.736-0.823)	
C_{MA-W}	0.640 (0.591-0.689)	2.97E-06
C_{MA-P}	0.644 (0.595-0.693)	5.78E-05
C_{GDM-PW}	0.649 (0.600-0.698)	8.88E-05
FGR		
C_{FGR-A}	0.773 (0.732-0.814)	
C_{FGR-W}	0.611 (0.563-0.658)	2.09E-06
C_{FGR-P}	0.616 (0.569-0.664)	8.62E-07
C_{FGR-PW}	0.621 (0.574-0.668)	9.24E-06
GDM		
C_{GDM-A}	0.718 (0.687-0.749)	
C_{GDM-W}	0.593 (0.559-0.627)	1.27E-07
C_{GDM-P}	0.618 (0.584-0.652)	4.86E-05
C_{GDM-PW}	0.626 (0.592-0.660)	1.31E-04
PE		
C_{PE-A}	0.825 (0.770-0.880)	
C_{PE-W}	0.690 (0.623-0.757)	4.68E-04
C_{PE-P}	0.696 (0.630-0.762)	3.37E-03
C_{PE-PW}	0.702 (0.636-0.768)	4.17E-03

P-values of AUC comparison were calculated using pROC package with default setting. 95% CI=95% confidence interval. MA = macrosomia. FGR = fetal growth restriction. GDM = gestational diabetes mellitus. PE = preeclampsia. P-value of AUC comparison between male- and female-bearing pregnancies was calculated using pROC package of R.

Supplemental Table S16. The performance of clinical features for predicting pregnancy complications

		AUC (95% CI)	Acc (%)	Sen (%)	Spe (%)	*P-value*
MA	$C_{MA\text{-}A}$	0.779 (0.736-0.823)	80.3	73.9	81.9	
	BMI	0.714 (0.668-0.761)	73.9	67.2	75.6	0.045
	Combination	0.762 (0.721-0.803)	73.4	80.7	71.6	0.32
FGR	$C_{FGR\text{-}A}$	0.773 (0.732-0.814)	78.9	74.6	80.0	
	BMI	0.690 (0.645-0.736)	74.9	59.2	78.8	9.32E-03
	Combination	0.786 (0.744-0.827)	81.8	73.1	84.0	0.311
GDM	$C_{GDM\text{-}A}$	0.718 (0.687-0.749)	72.1	71.2	72.4	
	BMI	0.540 (0.506-0.574)	58.7	44.6	63.4	9.66E-14
	Combination	0.682 (0.650-0.715)	68.1	68.5	67.9	2.20E-03
PE	$C_{PE\text{-}A}$	0.825 (0.770-0.880)	83.0	81.7	83.3	
	BMI	0.667 (0.598-0.735)	68.7	63.3	70.0	1.93E-04
	Combination	0.629 (0.560-0.699)	65.6	67.5	58.4	1

MA = macrosomia. FGR = fetal growth restriction. GDM = gestational diabetes mellitus. PE = preeclampsia. AUC = area under the receive operating characteristic curve. 95% CI = 95% confidence interval. Acc = accuracy. Sen = sensitivity. Spe = specificity. Combination meant that the BMI was taken as one of the features of set-A classifier of each pregnancy complication to develop novel classifier. *P-value* of AUC comparison was calculated by the pROC package of R. BMI means body mass index before pregnancy.

Supplemental Table S17. Functional annotation of genes in classifiers by retrieving literatures

Classifier	Gene	Function annotation	PMID
$C_{MA\text{-}A}$	SMC3	Central component of cohesin, a complex required for chromosome cohesion during the cell cycle.	18614053
	MASTL	Serine/threonine kinase that plays a key role in M phase by acting as a regulator of mitosis entry and maintenance.	20818157
	C1QTNF12	Insulin-sensitizing adipocyte-secreted protein that regulates glucose metabolism in liver and adipose tissue.	21849507

	MLXIP	It is involved in the regulation of genes in response to cellular glucose levels.	19706488
	MAP3K9	It plays a role in mitochondrial death signaling pathway, including the release cytochrome c, leading to apoptosis.	26573018
	APC2	This gene has its highest expression in the central nervous system and is involved in brain development through cytoskeletal regulation in neurons.	17367777
	NIPBL	It plays a role in developmental regulation.	15373324
	CREM	It is associated with Type 1 Diabetes Mellitus	17509149
C_{FGR-A}	CD63	Different levels of platelet activation in preeclamptic, normotensive pregnant, and non-pregnant women.	15118653
	TCF7	This gene is associated with type 1 diabetes.	12765974
	DNPEP	It likely to play an important role in intracellular protein and peptide metabolism.	28514442
	PTDSS2	The protein functions in cell signaling, blood coagulation, and apoptosis.	14984733
	VAV1	It couples tyrosine kinase signals with the activation of the Rho/Rac GTPases, thus leading to cell differentiation and/or proliferation.	21647562
	PNRC2	It plays roles in glucocorticoid receptor-mediated mRNA degradation by interacting with the glucocorticoid receptor NR3C1.	25775514
C_{GDM-A}	NAT10	It may affect microtubule stability and cell division.	19303003
	SIPA1	It affects cell cycle progression.	19765277
	WDR35	It promotes TNF-stimulated apoptosis.	20193664
	MICALL1	It may indirectly play a role in neurite outgrowth.	25086062
	CTNNB1	It regulates cell growth and adhesion between cells	28456466
	CLOCK	Polymorphisms in this gene may be associated with obesity and metabolic syndrome.	18541547
	BCKDHB	Mutations in this gene have been associated with maple syrup urine disease (MSUD), type 1B.	22326532
C_{PE-A}	NFKB2	It involves in inflammation and immune function, which is associated with preterm birth.	18818748
	AMOTL1	It induces the remodeling of the actin cytoskeleton.	19565639
	CDX1	It could play a role in the terminal differentiation of the intestine.	19059241

PMID = PubMed Unique Identifier.

Supplemental Table S18. Performance evaluation of the classifiers

Classifier	Positive predictive value	Negative predictive value
C_{MA-A}	0.483	0.927

C_{GFR-A}	0.506	0.926
C_{GDM-A}	0.462	0.883
C_{PE-A}	0.551	0.948
